# Escape response kinematics in two species of tropical shark: short escape latencies and high turning performance

**DOI:** 10.1242/jeb.243973

**Published:** 2022-11-18

**Authors:** José E. Trujillo, Ian Bouyoucos, William J. Rayment, Paolo Domenici, Serge Planes, Jodie L. Rummer, Bridie J. M. Allan

**Affiliations:** ^1^ Department of Marine Science, University of Otago, Dunedin 9016, New Zealand; ^2^ Australian Research Council Centre of Excellence for Coral Reef Studies, James Cook University, Townsville 4814, Australia; ^3^ PSL Research University, EPHE-UPVD-CNRS, USR 3278 CRIOBE, Université de Perpignan, 66100 Perpignan, France; ^4^ Department of Biological Sciences, University of Manitoba, Winnipeg, MB, R2T 2N2, Canada; ^5^ CNR-IAS, Località Sa Mardini, 09170 Torregrande, Oristano, Italy; ^6^ CNR-IBF, Area di Ricerca San Cataldo, Via G. Moruzzi N°1, 56124 Pisa, Italy; ^7^ Laboratoire d'Excellence CORAIL, EPHE, PSL Research University, UPVD, CNRS, USR 3278 CRIOBE, Papetoai 98729, French Polynesia; ^8^ Marine Biology, College of Science and Engineering, James Cook University, Townsville 4814, Australia

**Keywords:** Fast-starts, Vulnerability traits, Antipredator behaviour

## Abstract

Accelerative manoeuvres, such as fast-starts, are crucial for fish to avoid predation. Escape responses are fast-starts that include fundamental survival traits for prey that experience high predation pressure. However, no previous study has assessed escape performance in neonate tropical sharks. We quantitatively evaluated vulnerability traits of neonate tropical sharks by testing predictions on their fast-start escape performance. We predicted (1) high manoeuvrability, given their high flexibility, but (2) low propulsive locomotion owing to the drag costs associated with pectoral fin extension during escape responses. Further, based on previous work on dogfish, *Squalus suckleyi*, we predicted (3) long reaction times (as latencies longer than teleosts, >20 ms). We used two-dimensional, high-speed videography analysis of mechano-acoustically stimulated neonate blacktip reef shark, *Carcharhinus melanopterus* (*n*=12), and sicklefin lemon shark, *Negaprion acutidens* (*n*=8). Both species performed a characteristic C-start double-bend response (i.e. two body bends), but single-bend responses were only observed in *N. acutidens*. As predicted, neonate sharks showed high manoeuvrability with high turning rates and tight turning radii (3–11% of body length) but low propulsive performance (i.e. speed, acceleration and velocity) when compared with similar-sized teleosts and *S. suckleyi*. Contrary to expectations, escape latencies were <20 ms in both species, suggesting that the neurophysiological system of sharks when reacting to a predatory attack may not be limited to long response times. These results provide a quantitative assessment of survival traits in neonate tropical sharks that will be crucial for future studies that consider the vulnerability of these sharks to predation.

## Teaser

**Summary:** Tropical reef shark neonates show high manoeuvrability and short reaction times during antipredator escape responses compared with expectations based on previous work on teleost fishes and sharks.

## INTRODUCTION

Escape behaviours in teleost fishes have been well studied since the 1970s (see Domenici and Hale, 2019) as they include variables that are fundamental traits for fish survival. These behavioural traits are intrinsic of predator–prey interactions, defining its outcome (Domenici, 2010; Walker et al., 2005), thus indirectly affecting fitness (i.e. a functional trait; Schmitz, 2017; Violle et al., 2007). For example, many fish species employ a fast horizontal turn, rapidly changing direction to escape a predatory attack. These accelerative responses are termed fast-starts and occur in a broad range of aquatic species (Domenici and Hale, 2019). The most common motor pattern in fast-starts is the body bending in a ‘C’ shape (termed a C-start) as a result of a unilateral muscle contraction, which corresponds to stage 1 of the escape response (Domenici and Hale, 2019). Stage 1 is usually followed by an opposite body bend, referred to as stage 2 (Domenici and Blake, 1997). Depending on whether stage 2 is present, double-bend or single-bend responses can be defined (Domenici and Blake, 1997). Hence, fast-starts include essential functional traits linked to the prey's ability to escape (i.e. ‘vulnerability’ traits; Klecka and Boukal, 2013).

From an ecological perspective, some escape components (e.g. responsiveness, Fuiman et al., 2006; manoeuvrability, Walker et al., 2005; Webb, 1976a; locomotion, as speed and acceleration, Katzir and Camhi, 1993; Walker et al., 2005; escape latency, McCormick et al., 2018) are determinants of escape success, and hence, indicators of vulnerability to predation (Domenici and Hale, 2019). For example, sharks may seek refuge in discrete nearshore habitats where predation is assumed to be reduced (i.e. shark nursery grounds; Heithaus, 2007; Heupel et al., 2007). The shallows may offer refuge by physically excluding large predators (Guttridge et al., 2012), especially in tidally influenced environments (Wetherbee et al., 2007). Yet, several studies have reported high mortality rates in neonate sharks (Gruber et al., 2001; Heupel and Simpfendorfer, 2002; Manire and Gruber, 1993), which is most likely due to direct predation (Heupel et al., 2007). This view assumes that prey are passive victims, since it does not consider their ability to evade an attack (e.g. vulnerability traits). Hence, several components of the fast-start escape response can be fundamental vulnerability traits for neonate sharks experiencing high predation pressure. However, the components of the escape responses are poorly understood in chondrichthyans (i.e. sharks, rays and chimaeras), limiting our understanding of their vulnerability to predation during early life stages within ecologically relevant habitats.

Studying vulnerability traits (e.g. escape behaviours and their components) can help overcome our limitations to understand how neonate sharks exploit nearshore habitats with high predation pressure. However, despite the extensive work on escape behaviours in teleosts, there is a lack of detailed kinematic studies on taxa that include large individuals, such as chondrichthyans (but see Domenici et al., 2004; Seamone et al., 2014). This is most likely because sharks occupy high trophic levels and are therefore often seen as predators rather than prey (Estes et al., 2016; Ferreira et al., 2017), along with the myriad experimental constraints that come with testing larger animals. For example, neonates of blacktip reef (*Carcharhinus melanopterus*) and sicklefin lemon (*Negaprion acutidens*) sharks use very shallow (<1 m), nearshore habitats likely for predator avoidance (Bouyoucos et al., 2020; George et al., 2019). In fact, in Mo'orea, French Polynesia, the terrestrial reef flats are potential nurseries for both species (Bouyoucos et al., 2022). Nonetheless, there is little evidence that indicates that neonate sharks benefit from reduced predation risk in these shallow waters (e.g. see Baker and Sheaves, 2007). These two species are likely preyed on by adult conspecifics (J.E.T., personal observations) and other larger teleosts (e.g. potentially giant travelly *Caranx ignobilis*; McPherson et al., 2012). Several shark species that are confined in shallow nearshore habitats during early life are even vulnerable to predation by birds (e.g. see Russo, 2015). Consequently, it is likely that fast-start escape responses allow both species to exploit nearshore habitats around Mo'orea where predation is still a threat.

Several studies have focused on turning performance in sharks (Hoffmann and Porter, 2019; Hoffmann et al., 2019; Kajiura et al., 2003; Porter et al., 2011); yet, few have examined the turning kinematics (Domenici et al., 2004; Seamone et al., 2014) and latency (Schakmann et al., 2021) of shark escape responses. Domenici et al. (2004) showed that adult Pacific spiny dogfish (*Squalus suckleyi*; originally reported as *S. acanthias*; see Ebert et al., 2010) exhibit turns comparable to manoeuvre specialists (e.g. angelfish, *Pterophyllum eimikei*), but are slower in speed and acceleration (Domenici and Blake, 1991). High manoeuvrability but low locomotor performance in adult *S. suckleyi* was attributed to aspects of their body design (Domenici et al., 2004). Further studies have revealed that *S. suckleyi* modify their escape responses depending on predator size, speed and approach orientation (Seamone et al., 2014), indicating high plasticity in escape ability. Schakmann et al. (2021) found long latencies, averaging 97.8 ms after mechano-acoustic stimulation, in adult *S. suckleyi*, much longer than most teleosts. The authors suggested this is in line with previous histological observations that indicate that Mauthner neurons (M-cells) are absent in adult elasmobranchs (Bone, 1977), which typically control escape responses in most fishes (Eaton et al., 1977; Zottoli, 1977). Although typical escape latencies in teleosts range from 5 to 150 ms (Domenici and Hale, 2019), faster reaction times often confer a competitive advantage to the prey (McCormick et al., 2018), and M-cells usually reduce this latency (Eaton et al., 2001). Work on zebrafish *Danio rerio* shows that, by unilaterally removing one of the two Mauthner axons, escape latency increases from 8–10 ms to around 10–20 ms (Hecker et al., 2020). Our current knowledge suggest that M-cells are absent in adult elasmobranch specimens studied to date, such as *Mustelus vulgaris*, *Scyllium stellare*, *Scyliorhinus canicula*, *Raja punctata* and *Torpedo ocellata* (Bone, 1977; Stefanelli, 1980). However, a transient Mauthner apparatus is present in early ontogenetic stages of *Squalus acanthias* (embryos) and *Dalatias licha* (Bone, 1977).

Work on similar-sized teleosts and elasmobranchs predicts high turning agility, low locomotor performance and long escape latencies in neonate sharks. Experiments have shown that sharks are capable of tight turning radii (<10% of body length) and high turning rates (Domenici et al., 2004; Hoffmann and Porter, 2019; Kajiura et al., 2003; Porter et al., 2011). Although turning rate may be highly variable (Domenici et al., 2004), the length–turning rate relationship in aquatic vertebrates (fish and marine mammals; Domenici, 2001) predicts turning rates of around 783 and 695 deg s^−1^ for the indicative average sizes observed in neonate *C. melanopterus* (57.3 cm total length) and *N. acutidens* (66.5 cm total length) in Mo'orea, respectively. The high flexibility of the anterior part of the body (Kajiura et al., 2003) facilitates such high manoeuvrability (Aleyev, 1977). Indeed, turning performance is predicted by postural reconfiguration in sharks (Porter et al., 2011). However, pectoral fins increase drag and decelerate the shark during a turn (Hoffmann and Porter, 2019; Hoffmann et al., 2019; Hoffmann et al., 2020), contrary to teleosts, which typically press the pectoral fins against their body during an escape response, with some exceptions of pectoral fin extension (Domenici and Blake, 1997; Eaton et al., 1977). This drag-based turning mechanism likely limits propulsive performance (e.g. speed and acceleration) in sharks during an escape response, as observed in *S. suckleyi* (Domenici et al., 2004). With respect to the timing of the response, long escape latencies (>20 ms) are predicted for sharks owing to differences that are expected in the neural control mechanisms compared with teleosts and in line with recent evidence on adult *S. suckleyi* (Schakmann et al., 2021).

In this study, we examined the fast-start escape responses of neonate *C. melanopterus* and *N. acutidens* to quantitatively evaluate their vulnerability traits. We used mechano-acoustic stimulation and high-speed videography analysis of the startle responses to assess both non-locomotor and locomotor performance. We predicted performance based on the following hypotheses: compared with previous work on teleosts, neonate tropical sharks will show (1) high turning rates and tight turning radii, but (2) low propulsive performance (e.g. speed, acceleration and velocity) during fast-start escape responses, and (3) long escape latencies (>20 ms). We used indicative performance ranges for each variable reported in Domenici and Hale (2019), the data reported for *S. suckleyi* (Domenici et al., 2004; Schakmann et al., 2021), predictions from Domenici (2001), and data reported in the literature for similar-sized teleosts (e.g. pike *Esox lucius*, Harper and Blake, 1990; Frith and Blake, 1991; rainbow trout *Oncorhynchus mykiss*, Webb, 1976a,b; Harper and Blake, 1990).

## MATERIALS AND METHODS

All procedures were approved under James Cook University Animal Ethics Committee protocol A2394 based on the Australian government's guidelines for the care and use of animals for scientific purposes. Shark research in French Polynesia was approved under Arrêté no. 11491 issued by the Ministrère de la Promotion des Langues, de la Culture, de la Communication et de l'Environnement of the French Government on 16 October 2019 authorizing collection, possession and transportation of sharks and their tissues. These guidelines, from both Australian and French governments, were complementary and did not conflict with each other.

### Collections

Neonate blacktip reef sharks [*Carcharhinus melanopterus* (Quoy & Gaimard 1824), *n*=12] and sicklefin lemon sharks [*Negaprion acutidens* (Rüppell 1837), *n*=8] were collected between November 2019 and January 2020 around Mo'orea island (17°32′0″S, 149°50′0″W) in French Polynesia using monofilament gillnets (50×1.5 m with 5 cm mesh size) at dusk (17:00–20:00 h). Neonates were identified by their umbilical scar stage, which is used to estimate age classes (Chin et al., 2015; Weideli et al., 2019). Neonates were then transported by car in insulated coolers filled with aerated seawater to the Centre de Recherches Insulaires et Observatoire de l'Environnement (CRIOBE) (Bouyoucos et al., 2018). Total transport time was under 90 min before arriving at the CRIOBE facilities, and no injuries associated with this capture/transport method were recorded. Total length (*L*_T_) and body mass (*m*_b_) were 56.9±1.1 cm (mean±s.e.m.; range: 48.0 to 60.2 cm) and 0.98±0.02 kg in blacktip reef sharks, and 66.0±1.4 cm (range: 61.2 to 71.8 cm) and 1.31±0.07 kg in sicklefin lemon sharks, respectively. The morning following capture (day 1), sharks were marked with passive integrated transponder (PIT) tags (Biomark; www.biomark.com) inserted below the first dorsal fin for individual identification.

### Husbandry

Sharks were maintained in the laboratory for 9 days in 1250 l circular tanks (∼1.5 m diameter) with flow-through filtered seawater and aeration, and covered with 60% shade cloth. Blacktip reef sharks were maintained in groups of three, and sicklefin lemon sharks were maintained in groups of two because of their larger size. The open-sided facilities provided a natural photoperiod. Feeding started at day 2 in captivity. We fed sharks every second day with fresh tuna at 3–5% of body mass (Chin et al., 2015). To control for satiation, sharks were fasted for 48 h prior to experiments starting. Water was maintained at ambient temperature (26–29°C) similar to their natural daily temperature range (see Bouyoucos et al., 2021). At days 9 and 10 in captivity, water temperature was stabilized to 29.2±0.07°C. Escape trials took place on day 10 and sharks were release back to their original site of capture after a recovery period of 2–3 days (i.e. feeding resumed immediately after experiments and sharks remained undisturbed until release), using the same transporting procedures as for the collections, within the parturition season, and during the day, when predators are expected to be less active.

### Escape trail

**Fig. 1.**
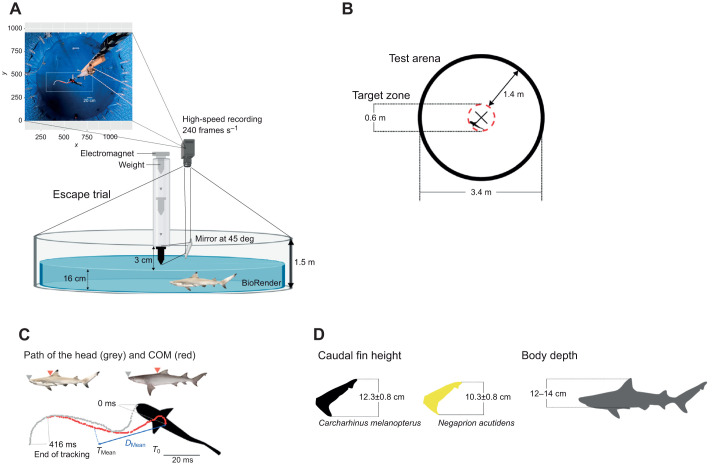
**Experimental set-up.** (A) Illustration of the pool (side view) showing the tapered steel weight falling through a PVC pipe. The moment the weight breaks the water (stimulus onset) is recorded using a mirror on the side of the pipe at 45 deg pointing the camera placed on a bird's-eye view perpendicular to the bottom of the pool (see top-left image inset). Two body landmarks are manually tracked from the still images of each frame (see C). Pool illustration is not to scale; image inset is to scale. (B) The test arena shows the target zone (red dashed circle) in relation to the size of the shark (black silhouette). The X marks the centre of the pool. (C) Body landmarks. Landmark positions are at 4.16 ms intervals. Displacement (*D*_Mean_, blue line) was measured from the position of the centre of mass (COM) at *T*_0_ until its position at *T*_Mean_. Distance travelled is same as the path of the COM (in red) but until *T*_1_ of each individual response. (D) Morphometrics. Shark illustrations by Erin Walsh.

On day 10, a single shark was randomly selected from the holding tank and transferred with a hand net to an adjacent 3.4 m diameter circular pool (referred to hereafter as the test arena, Fig. 1A; wall height ∼1.5 m) with the same blue coloration as the holding tanks. Transferer times were under 30 s. The test arena was filled with aerated, filtered seawater at the same temperature as the holding tank (29.2±0.07°C). To restrict movement in the vertical plane, water was maintained at a depth of 16 cm, which just allowed the whole body of the shark to be submerged (see Fig. 1D for caudal fin height and body depth reference). Before the escape trial, we allowed the shark to acclimate to the novel environment (the test arena) for 2 h. We determined this acclimation period based on ethograms obtained during pilot tests over a 3-h observation period (18 h of observation in total). We analysed average swimming speed and space use to control for variability in their activity level (the pre-startle state). See Fig. S1 for details on analysis and Fig. S2 for results. We also recorded spontaneous turns after acclimation, but before stimulation, to validate escape responses against un-stimulated turns (see Domenici et al., 2004).

We used a sudden (mechano-acoustic) stimulus to elicit a startle response because it has been shown to elicit stronger responses compared with other types of stimuli (Domenici and Hale, 2019). The stimulus consisted of a tapered steel weight (560 g) that was released by an electromagnet from 1.58 m above the test arena onto the surface of the water through a PVC pipe to avoid a premature response (Allan et al., 2014; Dadda et al., 2010). A mirror attached to the end of the pipe at 45 deg allowed visualization of the stimulus onset (Fig. 1A). Four reference lengths (20 cm long) were placed on the bottom of the test arena, around the impact point (i.e. at the same level of the shark). These were used for video calibration and to delimit the target zone. The target zone was 0.6 m in diameter and its edge was 1.4 m away from the wall (Fig. 1B). Sharks were only startled when they were within the target zone. Stimulus angle (SA) was 74.26±4.51 deg (range: 15.73–130.99 deg) and stimulus distance was 22.08±1.39 cm to the COM and 24.62±1.56 cm to the tip of the head. On no occasion did the stimulus touch the sharks or the bottom of the test arena. Each shark was stimulated at least three times and allowed to re-habituate for 20 min between each stimulation and its pre-startle state re-assessed. See timeline in Fig. S1 and results in Fig. S2. Each response was recorded at high speed (240 frames s^−1^) using a GoPro Hero7 Black camera with wide field of view (see section below for details on lens distortion correction). Three sharks were tested per day. To control for a potential temporal signal, trials took place during three different periods of the day, such that one individual was tested during the morning (9:00–11:00 h), one at midday (12:00–14:00 h) and one in the afternoon (15:00–18:00 h).

### Lens distortion correction and parallax effects

We used a GoPro Hero7 Black camera with a wide field of view (FOV) and the following video parameters: (1) equivalent focal length: 15–30 mm, (2) aspect ratio: 4:3, (3) pixel resolution: 1280×960, (4) vertical FOV: 94.4 deg, (5) horizontal FOV: 122.6 deg and (6) diagonal FOV: 149.2 deg. Prior to analysis, we used Adobe Premier Pro v22.2.0 to correct for wide-angle lens distortion. The lens distortion algorithm calibrated for GoPro cameras applied a –31 curvature correction to each video. This parameter is already established by the software as it is already calibrated for the camera model/lens type. For this reason, calibration parameters (e.g. *k*_1_, *k*_2_, *k*_3_, *k*_4_) cannot be accessed. The curvature of straight lines in the distorted image was rectified in the resulting undistorted image. This correction reduces the magnification effect of the centre of the image by reducing radial distortion. Tangential distortion (or de-centring distortion) was negligible. Parallax effects, in which a moving object appears to move a shorter or longer distance than it actually does, were also negligible because (1) we restricted the vertical movement of the sharks (see above), (2) we placed the reference lengths at the same level of the sharks, and (3) our camera was at the same angle and distance with respect to the bottom of the test arena throughout the trials. Finally, we exported corrected image sequences from the undistorted videos at 240 frames s^−1^ for escape trials and 120 frames s^−1^ for spontaneous turns.

### Video analysis

Two body landmarks were predefined for manual tracking purposes: the tip of the head and the origin of the first dorsal fin (see Fig. 1C). The second landmark was representative of the centre of mass (COM), based on the assumption that pre-first dorsal fin length, which is approximately 32% of *L*_T_ in *C. melanopterus* for 427–525 mm *L*_T_ individuals (Garrick, 1982), is similar to the calculated position of the COM in previous studies (e.g. 33% of *L*_T_ in *Squalus suckleyi*, Domenici et al., 2004; ∼35% of *L*_T_ in *Salmo gairdneri*, Webb, 1976a; 38% of *L*_T_ in *Clupea harengus*, Domenici and Batty, 1997).

We used the manual tracking plugin in ImageJ v2.0.0 for a two-dimensional video analysis frame by frame using the corrected image sequences. The corrected image resolution (1280×960 pixels) yielded ∼2.7 pixels cm^−1^. This resolution was enough for accurate visual analysis of zoomed frames when necessary (e.g. for estimation of latency). Using the reference lengths, a value of ∼0.3703 was applied for *x*-axis (forward displacement) and *y*-axis (lateral displacement) calibration. Calibration of the *z*-axis (vertical displacement) was not necessary, as water depth was sufficiently shallow to minimize shark movements in this plane. We digitized the *x*,*y* coordinates (tracking points) of the predetermined body landmarks. Distance between tracking points and instantaneous speed (between two successive frames) were obtained from the tracking software. For the escape responses, tracking was initiated one frame before the first detectable movement of the tip of the head and continued for 100 frames (i.e. 416 ms). For spontaneous turns, tracking started one frame before the first detectable movement of the head and finished with the end of the turn. Tracking coordinates were then imported to the R environment for further analysis (see below).

### Responsiveness, directionality and escape latency

Responsiveness was measured as the proportion of escape responses out of the total number of stimulations for each shark. Directionality was defined as the proportion of responses in which the body bent in a direction away from the stimulus (i.e. ‘away responses’ as opposed to ‘towards responses’) out of the total number of responses, and did not necessarily indicate the final escape trajectory (Domenici et al., 2011). Escape latency was measured as the time between stimulation and the onset of the escape response (as the first detectable movement of the head) in milliseconds for all successful escape responses in which the weight was visible in the mirror (see Fig. 1A). We then calculated the mean and minimum latencies for each individual (Schakmann et al., 2021).

### Durations and stages

Stage 1 (S1) and stage 2 (S2) were defined by the turning of the anterior segment of the body (tip of head to COM; Domenici et al., 2004; Kasapi et al., 1993). These are body bends in opposite directions. The time taken to form each body bend corresponded to stage 1 and stage 2 durations (*T*_S1_ and *T*_S2_), respectively. To define *T*_S1_ and *T*_S2_, we used the smoothed turning rate versus time curve (see below) and applied the function uniroot.all from the package stats (https://www.r-project.org/) to find the time points defining the stages. The first time point (*T*_0_), at the start of the tracking, was one frame before the first detectable movement of the head. Stage 1 started at *T*_0_ and finished with a reversal of turn direction, at *T*_1_, indicated by the smoothed turning rate curve crossing 0 deg s^−1^. Stage 2 started at *T*_1_ and finished with a stop or reversal of turning direction, at *T*_2_; that is, when the turning rate curve crossed 0 deg s^−1^ again. Total escape response duration (*T*_Escape_) was the sum of *T*_S1_ and *T*_S2_. The mean duration of all responses recorded, by species, was defined as *T*_Mean_. Although variable, continuous swimming or coasting have been defined as the final stage (S3) in some fish species (Rand and Lauder, 1981; Webb and Skadsen, 1980; Weihs, 1973). Here, stage 3 started at *T*_2_, and although the end was not defined, we used the tracking points until frame 100 as an arbitrary time frame for stage 3 analysis (see below). Turning rate data paired with video inspections were used for stage 3 analysis.

### Turning kinematics: angles, turning radius and turning rate

Angles were measured using the angle tool in ImageJ as the relative positions of the line projecting from the tip of the head to the COM between the start and end of each stage (stage 1 angle: θ_S1_; stage 2 angle: θ_S2_). The rotation of this line was measured in degrees. Because stage 2 corresponds to a body bend opposite to that of stage 1, θ_S2_ bears a negative sign.

Turning rate was defined as the angular velocity of the anterior segment of the body (tip of head to COM) in deg s^−1^. The maximum turning rate for each stage was extracted from smoothed turning rate data (ω_S1_ and ω_S2_, respectively; see ‘Data processing and statistical analyses’ for details on smoothing methods). Mean turning rate was obtained as the ratio between angle and duration for stage 1 (ω_Mean_; Domenici et al., 2004).

Turning radius (*R*_Turn_) was calculated using the formula in Domenici and Blake (1991):
(1)

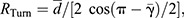


where 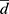 is the mean instantaneous distance moved (see below) and 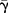 is the mean instantaneous angle of turn of the COM during stage 1. The result was divided by *L*_T_, as it has been demonstrated to be proportional to body length (Howland, 1974; Webb, 1976a).

We also measured turning angle, duration and mean turning rate using image sequences (at 120 frames s^−1^) of spontaneous turns during routine swimming for unstimulated individuals for both species.

### Distance–time variables: distance, displacement, velocity, speed and acceleration

We measured two different distance variables: distance travelled and displacement. We used a fixed time (*T*_Mean_) to measure these variables to avoid any performance bias owing to differences in escape duration (Domenici and Blake, 1991; Domenici et al., 2004; Webb, 1976b). The cumulative distance travelled (DT_Mean_) was calculated as the length of the path of the COM in metres from *T*_0_ until *T*_Mean_. Displacement (*D*_Mean_; m) was measured as a straight line between the positions of the COM at *T*_0_ and at *T*_Mean_ (see Fig. 1C). Velocity (ν; m s^−1^) was obtained by dividing *D*_Mean_ by *T*_Mean_. Average speed (*U*_Avg_, m s^−1^), maximum speed (*U*_Max_; m s^−1^) and maximum acceleration (α_Max_; m s^−2^) were calculated from the smoothed distance–time data obtained from ImageJ during the escape response (i.e. *T*_Escape_; see ‘Data processing and statistical analyses’ for details on smoothing methods). Velocity differed from speed (*U*_Avg_ and *U*_Max_), as the former was based on how fast the shark displaced its body from point A (one frame before stimulation) to point B (*T*_Mean_), and the latter was derived from the path of the COM throughout the escape sequence (*T*_Escape_).

### Data processing and statistical analyses

All data processing and statistical analyses were performed in the R statistical environment (https://www.r-project.org/). For the analysis of turning rate, speed and acceleration, we used smoothed curves for each of these variables. We applied a locally estimated scatterplot smoothing (LOESS) to the raw data using the loess function. The degree of the polynomial (1 or 2) and the size of the neighbourhood (α) were optimized using a 5-fold cross-validation procedure specific to each escape response using the kfold function from the dismo package (https://CRAN.R-project.org/package=dismo). The root mean square error (RMSE) was obtained from the cross-validation results for each model combination with the function rmse from the package hydroGOF (https://CRAN.R-project.org/package=hydroGOF) such that the fit with the minimum RMSE was used for smoothing.

Because each individual was stimulated at least three times, we checked differences between stimulations using a one-way ANOVA using complete cases from blacktip reef shark data. We found no significant differences for turning radius (*F*_2,28_=0.56, *P*=0.60), *T*_S1_ (*F*_2,29_=0.84, *P*=0.44), *T*_S2_ (*F*_2,24_=0.17, *P*=0.84), *T*_Escape_ (*F*_2,24_=1.23, *P*=0.31), ω_S1_ (*F*_2,29_=1.24, *P*=0.30) or ω_S2_ (*F*_2,25_=0.35, *P*=0.71) between stimulations in neonate blacktip reef sharks. This indicated no habituation effect or muscle fatigue over the consecutive stimulations (Marras et al., 2011). Hence, all responses were used in the results. The smaller sample size and unbalanced structure of the data for sicklefin lemons sharks precluded a similar analysis for this species. However, our results for blacktip reef sharks and results from other studies (i.e. Marras et al., 2011) were sufficient evidence to also pool all the data for sicklefin lemon sharks.

We pooled all data for summary results but used linear mixed-effects models (LMM) for all regressions to account for the non-independence owing to the repeated measures in the data. This approach allowed us to also account for the unbalance structure of the data (i.e. not all sharks had three startles). We used the lme function from the nlme package (https://CRAN.R-project.org/package=nlme) and included the stimulus number as a random effect (three levels as we performed three stimulations for each individual). To investigate the relationship between turning performance and locomotor performance, we built LMMs with velocity (m s^−1^) as the dependent variable with either θ_S1_,θ_S2_, ω_S1_ or ω_S2_ as the explanatory (fixed effect) variable. Stimulus number was included as a random effect. Model validating was done via visual inspection of residuals following Zuur et al. (2009).

## RESULTS

Individuals were deemed acclimated to the test arena after 2 h, as evidenced by undisturbed swimming. Sharks were startled three times, 20 min after each stimulation. Normal swimming activity resumed within the 20 min (see results in Fig. S2). On average, blacktip reef sharks were swimming at 0.36±0.01 m s^−1^ and sicklefin lemon sharks at 0.46±0.03 m s^−1^ before being startled. Average swimming speed was not significantly different between pre-startle states in blacktip reef sharks (one-way ANOVA: *F*_1,30_=3.9, *P*=0.06) or in sicklefin lemon sharks (one-way ANOVA: *F*_1,12_=2.8, *P*=0.12). All individuals resumed similar space use following a startle, and space use was not significantly different between pre-startle states in either species (see one-way ANOVA results in Fig. S2). Blacktip reef sharks spent 85.7±2.7% of the time in open spaces, whereas sicklefin lemon sharks divided their time almost equally between areas (percentage time in open space: 50.2±3.6%). This difference in space use was statistically significant between species (one-way ANOVA: *F*_1,43_=57.6, *P*<0.05).

**Fig. 2.**
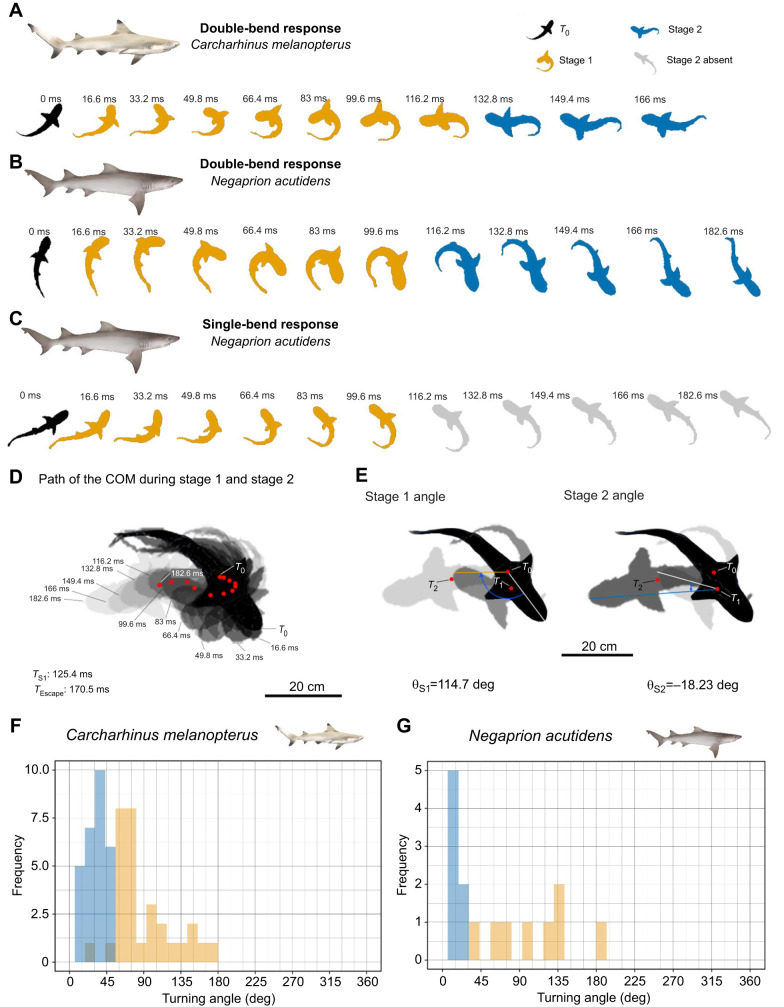
**Escape response sequences, COM positions and turning angles.** Silhouettes at intervals of 16.6 ms of (A) neonate *Carcharhinus melanopterus* and (B) neonate *Negaprion acutidens* double-bends and (C) a neonate *N. acutidens* single-bend. Not to scale. (D) Superimposed silhouettes showing the path of the COM (red dots) during stages 1 and 2 at 16.6 ms intervals in a *C. melanopterus* double-bend response and its (E) corresponding stage 1 and stage 2 turning angles. Angles are measured between the lines projecting from the COM to the tip of the head, at the start of each stage (white) until *T*_1_ for stage 1 (orange line), or until *T*_2_ for stage 2 (blue line). (F) Frequency distributions of absolute turning angles in degrees during stage 1 (orange) and stage 2 (blue) for both species. Angles do not indicate final trajectory of escape. Shark illustrations by Erin Walsh.

We were able to successfully video record 32 responses from 12 blacktip reef sharks and 20 responses from seven sicklefin lemons sharks. Both species responded to the mechano-acoustic stimulus with a fast unilateral contraction bending the body to one side, followed by a body bend in the opposite direction (see escape sequences in Fig. 2A,B). However, two responses recorded in sicklefin lemon sharks were not fast-starts and were removed from analysis. Responsiveness was 100% for blacktip reef sharks (32 out of 32 responses) and 64.6% for sicklefin lemon sharks (11 out of 18 responses). This difference in responsiveness between the two species was statistically significant (one-way ANOVA: *F*_1,18_=7.98, *P*<0.05). Only one escape response, and only in blacktip reef sharks, was towards the weight, such that similar directionality (97% and 100%) was observed in blacktip reef sharks and sicklefin lemon sharks, respectively.

**Fig. 3.**
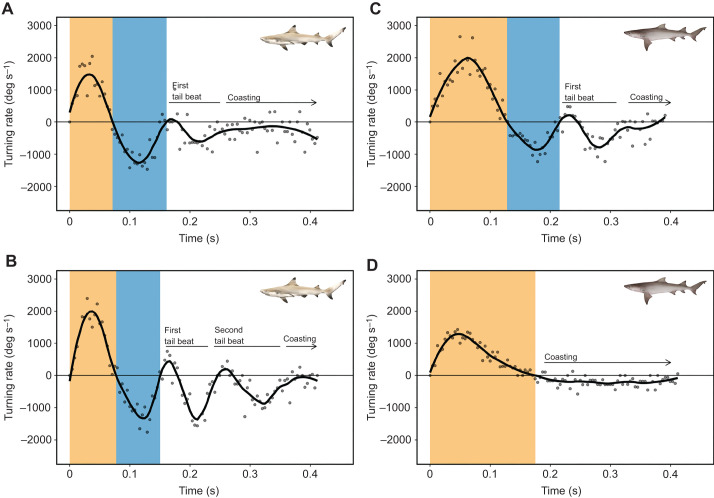
**Turning rate.** Four individual escape responses showing a neonate *Carcharhinus melanopterus* double-bend response with (A) one and (B) two tail beats followed by coasting during stage 3, and neonate *Negaprion acutidens* (C) double-bend and (D) single-bend responses. Stage 3 starts when stage 2 ends, but stage 3 end is undefined. Black solid curves are optimized LOESS smooth (see Materials and Methods). Dots are raw turning rate data. Shaded areas represent stage 1 (orange) and stage 2 (blue) durations. Shark illustrations by Erin Walsh.

The first whole-body bend had a characteristic C-shape (i.e. stage 1, Domenici and Blake, 1997). The second, contra-lateral body bend, clearly defined from changes in the direction of the head, constitute stage 2 (*sensu* Domenici and Blake, 1997). These double-bend escape responses are illustrated in the turning rate data (Fig. 3A–C) with the first positive peak representing the formation of the C-shape and a subsequent depression with negative values representing the opposite body bend. All escape responses in blacktip reef sharks were double-bends (*n*=32 escape response). In sicklefin lemon sharks, the turning rate data of 11 escape responses showed that eight responses were double-bends and three of them were single-bends (Fig. 2C and Fig. 3D). We have therefore described double-bends separately from single-bend responses in sicklefin lemon sharks.

### Escape latency

**Fig. 4.**
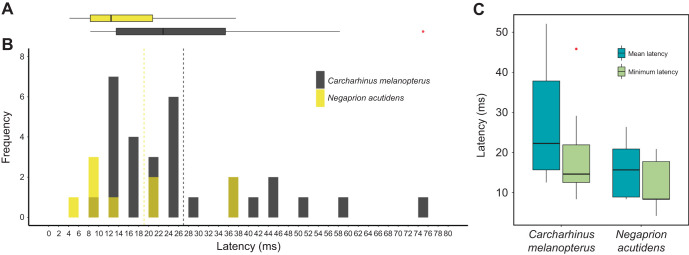
**Latency in milliseconds in neonates of two tropical shark species.** (A,B) Latency frequency distribution and associated boxplot for neonate *Carcharhinus melanopterus* (black, *n*=30 responses) and neonate *Negaprion acutidens* (yellow, *n*=9 responses). Vertical black dashed lines are the means. (C) Comparison between mean and minimum escape latencies for both species. Mean and minimum latencies were not significantly different between species (see ‘Escape latency’ section in Results). Boxplots show median (solid black bar), first and third quartiles (left and right hinges, respectively), lowest and maximum values (left and right whiskers, respectively), and extreme values (red dots). Single- and double-bends are combined for *N. acutidens*.

Escape latency ranged from 8.33 to 75.0 ms (*n*=30 responses; Fig. 4A,B) in blacktip reef sharks and from 4.17 to 37.5 ms (*n*=9 responses; Fig. 4A,B) in sicklefin lemon sharks. Latency values were less than 20 ms in 40% of the responses in blacktip reef sharks and in 55% of the responses in sicklefin lemon sharks. The three single-bend responses in sicklefin lemon sharks had latencies of 8.33, 20.8 and 37.5 ms (all different individuals). We observed a peak in frequency that was below the 20 ms mark in both species, that is at 13 ms in blacktip reef sharks and at 9 ms in sicklefin lemon sharks (Fig. 4B). Mean latency – the mean of all successful trials for a single individual – was 27.9±4.96 ms in blacktip reef sharks (*n*=12 individuals) and 15.9±3.18 ms in sicklefin lemon sharks (*n*=6 individuals; Fig. 4C). On average, minimum latency – the shortest latency an individual achieved across all its successful trials – was 19.4±3.74 ms in blacktip reef sharks (*n*=12 individuals) and 11.8±2.93 ms in sicklefin lemon sharks (*n*=6 individuals). We found no significant differences in mean (one-way ANOVA: *F*_1,16_=3.2, *P*=0.093) or minimum latencies (one-way ANOVA: *F*_1,16_=2.34, *P*=0.146) between blacktip reef sharks and sicklefin lemon sharks (Fig. 4C).

### Double-bend response kinematics

**Fig. 5.**
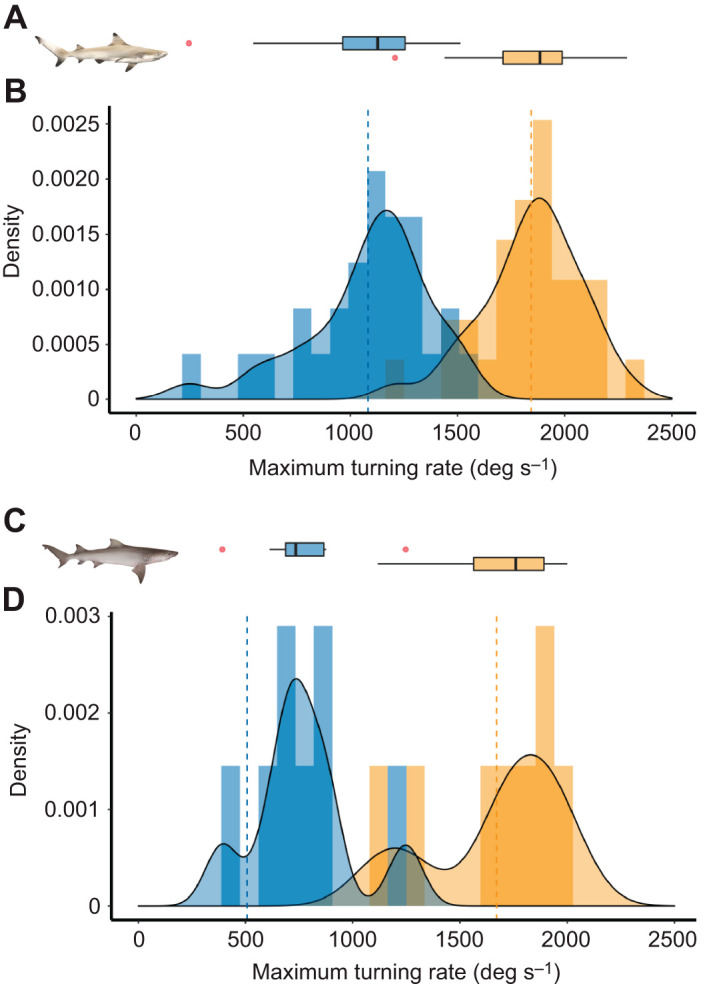
**Absolute maximum turning rate density distributions for double-bend escape responses.** (A,B) *Carcharhinus melanopterus* and (C,D) *Negaprion acutidens*. Orange, stage 1; blue, stage 2. Smoothed distributions are kernel density estimates. Vertical dashed lines are means. Associated boxplots (A,C) show median (solid black bar), first and third quartiles (left and right hinges, respectively), and lowest and maximum values (left and right whiskers, respectively), and extreme values (red dots). Shark illustrations by Erin Walsh.

During their first body bend (stage 1), both species showed fast angular velocities (i.e. ω_S1_ and ω_Mean_; Table 1). Angular velocities observed during stage 2 (ω_S2_) in blacktip reef sharks had a much broader range than in sicklefin lemon sharks (Fig. 5). It is important to note, however, that the sample size for the sicklefin lemon sharks was smaller than that for the blacktip reef sharks. Both species had a wide range of stage 1 turning angles (Fig. 2F,G), averaging around 90 deg (Table 1) but the maximum observed were relatively large (Table S1). Angles of turn during the second body bend (θ_S2_) were smaller (Table 1) and with a narrower range (Fig. 2F,G). The first body bend had a tight turning radius, the smallest reported (that is from a single event) was 0.01*L* in both species. The frequency distribution of turning radius was skewed toward values that were lower than the mean in both species (Fig. S3). During spontaneous turns, both species turned with slower angular velocities (ω_Mean_) at smaller turning angles (θ_S1_) than escape responses (Tables S1 and S2).

**Table 1.**
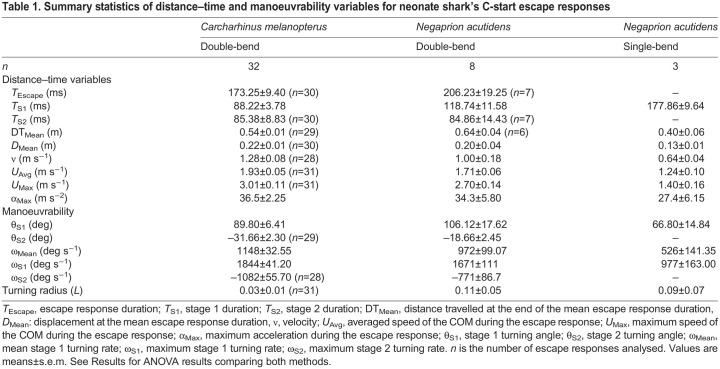
Summary statistics of distance–time and manoeuvrability variables for neonate shark's C-start escape responses

**Fig. 6.**
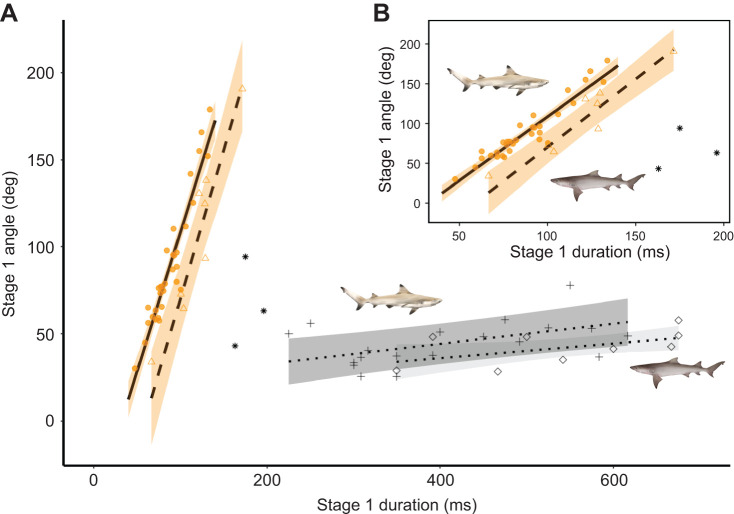
**Stage 1 duration versus angle for escape and routine turns.** (A) Double-bend escape responses of *Carcharhinus melanopterus* (circle, solid line) and *Negaprion acutidens* (triangle, dashed line), and routi­ne turns of *C. melanopterus* (cross, dotted line) and *N. acutidens* (diamonds, dotted line). (B) Close-up of escape responses only. Black asterisks are single-bend escape responses in *N. acutidens*. Fits are linear mixed-effects regressions. Bands are 95% confidence intervals. Observations are individual escape responses or routine turns. See Table S4 for model results. Orange, stage 1; grey, routine turns. Shark illustrations by Erin Walsh.

All durations (*T*_Escape_, *T*_S1_ and *T*_S2_) are summarized in Table 1, and frequency distributions are shown in Fig. S3. The escape response duration (*T*_Escape_) was comparable to that of *S. suckleyi* but longer compared with similar-sized teleost species (Table S1). Longer durations were observed during spontaneous turns in both species (Tables S1 and S2). There was no significant effect of *L*_T_ on *T*_S1_ (LMM: *F*_1,30_=0.273, *P*=0.605, *R*^2^=−0.02) or *T*_Escape_ (LMM: *F*_1,30_=0.001, *P*=0.9734, *R*^2^=−0.03) in blacktip reef sharks. Similarly, *L*_T_ did not affect *T*_S1_ (LMM: *F*_1,6_=0.33, *P*=0.587, *R*^2^=−0.106) or *T*_Escape_ (LMM: *F*_(1,6)_=0.56, *P*=0.482, *R*^2^=−0.067) in sicklefin lemon sharks. *T*_S1_ was linearly correlated with the angle of turn during stage 1 (θ_S1_) in both species (Fig. 6A,B and Table S4). Double-bend escape responses occupied a different section of the graph in Fig. 6A compared with spontaneous turns, for both species.

The COM reached maximum speeds right at the end or after stage 2 (e.g. Fig. 8). Then, the COM exhibited a slight decrease in speed before 416.66 ms (i.e. end of the tracking sequence). Both species reached similar speeds during the escape response (Table 1), that were comparable to those of similar-sized teleosts, but higher than that of *S. suckleyi* (Table S1). *U*_Max_ did not correlate with ω_S1_ (LMM: *F*_1,25_=3.16, *P*=0.09, *R*^2^=0.07) or ω_S2_ (LMM: *F*_1,25_=0.015, *P*=0.90, *R*^2^=−0.04) in blacktip reef sharks. Unfortunately, the lower sample size in sicklefin lemon sharks precluded the possibility to draw a reliable correlation. Peak acceleration occurred at the beginning of the first stage, and it was similar in both species (see Table 1) but decreased during the escape response (not shown).

**Fig. 7.**
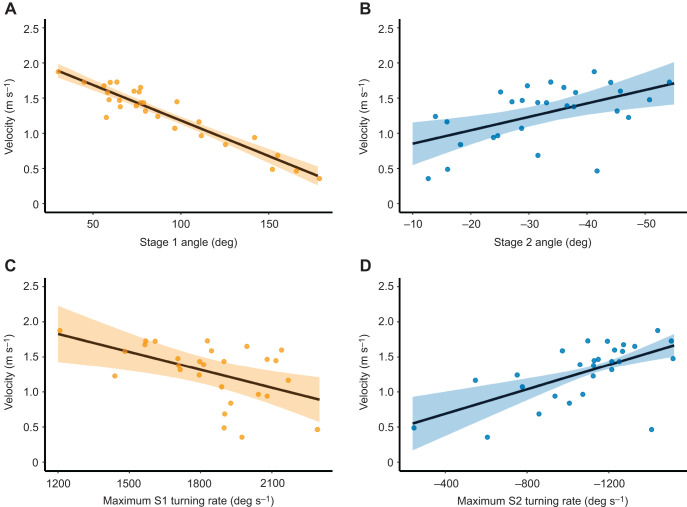
**Linear mixed-effects fits between turning performance and locomotor performance in neonate *Carcharhinus melanopterus*.** (A,B) Angles versus velocity and (C,D) maximum turning rates versus velocity. Velocity was measured at the mean escape response duration. Orange, stage 1; blue, stage 2. Bands are 95% confidence intervals. Correlations were built with absolute values for stage 2 (negative sign only indicates direction of bend relative to stage 1). Observations are individual escape responses. See Table S4 for model results.

In a straight line, both species displaced away from their initial position by a similar distance (Table 1). The cumulative distance travelled by the path of the COM (at the mean escape response duration) was comparable in both species (Table 1; see Fig. 2D for an example of the path of the COM). In line with the above, both species displaced with a similar velocity (ν; Table 1). In blacktip reef sharks, ν was negatively affected by the θ_S1_. When the angle of the turn during stage 1 was large, sharks displaced more slowly (Fig. 7A). In contrast to θ_S1_, θ_S2_ was positively correlated with velocity (Fig. 7B). Note that these correlations were built using velocity measured at *T*_Mean_, such that there is no effect of the longer duration of turns with larger angles.

### Single-bend response kinematics

**Fig. 8.**
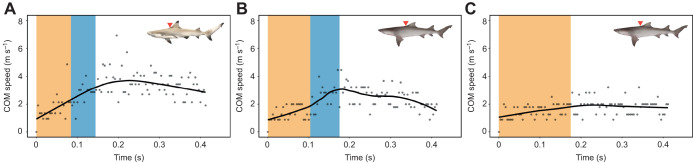
**Speed of the COM throughout the escape response.** Examples of double-bend escape responses for (A) *Carcharhinus melanopterus* and (B) *Negaprion acutidens*, and (C) single-bend escape response for *N. acutidens*. Black solid curves are optimized LOESS smooth (see Materials and Methods). Dots are raw speed data. Shaded areas represent stage 1 (orange) and stage 2 (blue) durations. Tracking landmark (i.e. COM) is indicated by the red triangle. Shark illustrations by Erin Walsh.

Single-bend responses were only observed in three sicklefin lemon shark individuals and not in blacktip reef sharks. In all cases, stage 1 was followed by coasting and no stage 2 was visible in the turning rate data (Fig. 3D). Single-bend kinematics are summarized in Table 1. We were unable to obtain a reliable correlation between *T*_S1_ and θ_S1_ owing to the lower number of single-bends (*n*=3). However, all single-bend responses occupied an area in the graph closer to that of double-bend escape responses and not to routine turns (Fig. 6). Therefore, these three responses were clearly of a higher intensity than routine turns, but less intense than double-bends. For example, *U*_Max_ was not much higher than *U*_Avg_ in single-bend responses (Fig. 8E and Table 1) and the fastest of all single-bend responses reached a *U*_Max_ of only 1.70 m s^−1^ (62 cm *L*_T_ individual). Displacement and velocity were calculated at the mean *T*_S1_ (Table 1). Velocity was 2 and 1.6 times slower than blacktip reef and sicklefin lemon sharks' double-bends, respectively. Unfortunately, the low number of single-bends (*n*=3) precluded the possibility to statistically test for differences between single-bends and double-bends in sicklefin lemon sharks. See Table S3 for data on all escape responses.

### Stage 3

We observed a consistent pattern in the motor characteristics that followed stage 2 in double-bend responses. This pattern (defined as stage 3) was observed and analysed in 31 and 5 responses in blacktip reef and sicklefin lemon sharks, respectively. In the remaining responses, we were unable to determine whether stage 3 was present as the sharks were in a blind spot for the camera during this section of the tracking. One or two tail beats were followed by coasting, in which the tail aligned with the midline during stage 3. This pattern was defined in the turning rate data by a positive signal followed by a negative-going signal of similar intensity (Fig. 3A–C). The start of stage 3 was marked by the first positive signal at the end of stage 2 (Fig. 3A–C). The end of stage 3 was undefined. However, tail beats were always completed, so that the tail aligned with the midline (and coasting followed), before 416.66 ms (i.e. before 100 frames), regardless of whether there were one or two tail beats. Notably, speed did not change, as no peaks were observed in the speed curve at this stage, regardless of whether these tail beats were present.

## DISCUSSION

Our results allowed us to quantitatively assess the escape performance of neonate sharks through several components (vulnerability traits) of their escape behaviour. We used neonates of two highly mobile tropical reef shark species and successfully obtained escape kinematic data under laboratory conditions. Both species engaged in fast-start escape responses typical of those observed previously in teleosts and in adult *S. suckleyi* (Domenici et al., 2004). Specifically, and for the first time in neonate tropical reef sharks, we describe C-start single-bend and double-bend escape responses. Our results showed that neonates of both species of tropical shark had higher than predicted turning rates (1671–1844 deg s^−1^), and tight turning radii (3–11% of body length). This is in line with a high turning manoeuvrability (hypothesis 1; see Introduction). We also showed that speeds (4.1–5.2 BL s^−1^) and accelerations (34.3–36.5 m s^−2^) were at the lower end for both species, when compared with published data in similar size species of teleosts, in line with hypothesis 2. Contrary to our expectations for shark reaction times (>20 ms, hypothesis 3), we reported short escape latencies (<20 ms). Finally, we emphasize the relevance of a third stage (stage 3), rarely described before (Weihs, 1973), to characterize the escape ability in sharks (i.e. mainly for propulsive performance) beyond the commonly studied kinematic stages in escape responses. Our results are relevant to understand how neonate sharks may exploit nearshore habitats where predation pressure is high.

High turning performance, based upon high turning rates, tight turning radius and large maximum turning angles, was observed in neonates of both species of sharks. Both species also exceeded theoretical expectations (783 and 695 deg s^−1^ expected values for 57.3 and 66.5 cm long individuals, respectively; versus 1148 and 972 deg s^−1^ observed in blacktip and sicklefin lemon sharks, respectively) based on the length-turning rate relationship in aquatic vertebrates (fish and marine mammals; Domenici, 2001). In contrast to previous work on *S. suckleyi* and similar-sized teleosts, our study demonstrated that the turning rate (ω_Mean_) for double-bend responses was 2.43 (blacktip reef shark) and 2.06 (sicklefin lemon shark) times higher than that of fast responses reported for adult *S. suckleyi* of a similar length (Domenici et al., 2004). This difference may be due to the difference in ambient temperature, which differed greatly between this study (29°C) and Domenici et al. (2004) (12°C). Indeed, other traits associated with turning performance, namely muscular contraction time (Wakeling, 2005), flexibility (Domenici, 2001) and postural reconfiguration (Porter et al., 2011), are likely to be affected by temperature. Unfortunately, we were not able to measure these traits in either species. Large maximum turning angles are also a characteristic of flexible species (Domenici, 2001) and typically range from 0 to 180 deg (Domenici and Hale, 2019). Neonate tropical sharks reached turning angles as large as 178.9 deg (blacktip reef shark double-bends), 138.28 deg (sicklefin lemon shark double-bends) and 190.7 deg (sicklefin lemon shark single-bends). This ability to turn with large angles, combined with a large range of escape angles, is a potential advantage as this will allow for a wide range of escape trajectories, independent of the angle of the approaching predator (Domenici and Hale, 2019; Domenici et al., 2011).

Another characteristic of high manoeuvrability was the tight turning radius observed in both species (3–11% of body length), equivalent to turning specialists (∼6%; Domenici and Hale, 2019), and in contrast to the larger turning radius observed in cruising specialists with rigid bodies such as yellowfin tuna, *Thunnus albacares* (∼40%; Blake et al., 1995). Tight turning radii can be used by prey animals for their advantage (Weihs and Webb, 1984) and can be a key parameter in determining the outcome of predator–prey interactions (Webb, 1976a). We observed asynchronous movements of pectoral fins in both species studied here, like in *S. suckleyi* (Domenici et al., 2004) and in unstimulated juvenile bonnethead sharks, *Sphyrna tiburo* (Kajiura et al., 2003). Therefore, it is possible that *C. melanopterus* and *N. acutidens* use pectoral fin extension to reduce turning radius as suggested by Domenici and Blake (1997). Pectoral fin activation seems to occur even a few milliseconds before any detectable movement of the head in *C. melanopterus* and *N. acutidens* (J.E.T., personal observations). Pectoral fins are likely an important control surface for shark escape responses. Indeed, Kajiura et al. (2003) found that *S. tiburo* reduced turning radius using the pectoral fin inside the body curvature as a pivot point, unlike juvenile sandbar *Carcharhinus plumbeus* and scalloped hammerhead *Sphyrna lewini*. This high manoeuvrability will be advantageous in structurally complex environments, such as coral reefs. Further, high manoeuvrability represents a competitive advantage for neonate sharks over larger predators (e.g. potentially larger individuals of the same species or closely related species; J.E.T., personal observations), because turning performance decreases with body size (Wakeling, 2005). Taken together, these results suggest that neonate tropical reef sharks can outmanoeuvre their larger predators, as they are capable of rapid changes in direction, a wide range of escape trajectories, and tight turns in narrow spaces.

It is also possible that the extent to which certain mechanisms that increase turning performance used by the sharks (e.g. pectoral fins acting as rudders) may cause a relatively low locomotor performance owing to the considerable drag costs associated (Domenici and Blake, 1997). In fact, other species of shark were observed to actively rotate pectoral fins, which increases drag during a turn, but this causes a deceleration (Hoffmann and Porter, 2019; Hoffmann et al., 2019; Hoffmann et al., 2020). When compared with previous work on teleost species, propulsive performance in tropical neonate sharks appears to be relatively low compared with most teleosts of a similar size. Although speed and acceleration were higher in *C. melanopter*us and *N. acutidens* compared with *S. suckleyi*, such differences may be attributable to difference in temperature (29°C compared with 12°C for *S. suckleyi*). However, speed and acceleration in neonate sharks was in the lower end of the indicative range for these parameters in teleosts (see Domenici and Hale, 2019), despite the considerable higher temperature in this study compared with those used in previous studies on teleosts. Interestingly, most teleosts press their pectoral fins against the body during escape responses (with some exceptions; Domenici and Blake, 1997) and some ‘accelerator’ specialists (e.g. *Esox lucius*) can achieve both high manoeuvrability and acceleration. Hence, future studies may need to focus on addressing the root causes for the low propulsive performance observed so far in the escape responses of sharks when compared with teleosts.

The high manoeuvrability during stage 1 may lead to a reduction in velocity in tropical neonate sharks as in many other teleosts (Domenici and Blake, 1991; Domenici and Blake, 1993). This trade-off is not restricted to sharks or teleosts (e.g. see Wynn et al., 2015) and can in part be compensated by stage 2 kinematics in tropical neonate sharks. Stage 2 corresponded to the main propulsive phase in neonate sharks as in teleosts (Domenici and Hale, 2019). A large turning angle and high turning rate during stage 2 was associated with an increase in velocity. Therefore, although an increase in the rate of body bending during stage 1 can cause a decrease in muscle force and power production (Wakeling et al., 1999), the tail sweep after stage 2 may be an important source of thrust in tropical neonate sharks. In fact, thrust can increase with postural curvature (Turesson et al., 2009). Experiments on escape success paired with fast-start kinematics would be needed to investigate the effect of different escape kinematics on vulnerability to predation. Another method of partial compensation for low locomotor performance may result from the power stroke of stage 3 tail beats. Because sharks are unable to generate forward thrust by paddling their pectoral fins (Wilga and Lauder, 2000, 2001, 2004), propulsion is mainly the result of the rearward propagating wave of the body curvature. Therefore, extra thrust generated by the tail beats observed during stage 3 can generate an increase in speed but may incur higher energetic costs. Undulatory motions (e.g. both from stage 2 and 3) seem to be an important source of thrust for neonate tropical sharks escape responses. Hence, it would be relevant to investigate the role of undulatory reconfiguration (Long et al., 2010) in escape success in sharks.

Escape responses are energetically expensive and, therefore, overall escape performance must meet a compromise with energy expenditure. For example, escapes lacking stage 2 (i.e. single-bend responses) may be less energetically costly to undertake than double-bend responses (Domenici and Hale, 2019). In fact, under challenging environmental conditions, such as hypoxia (Domenici et al., 2007) or increased temperature (Allan et al., 2015), or during physiological challenges such as starvation (Yan et al., 2015), single-bend responses are common and more frequent (Lefrançois et al., 2005). We found a broad range of ω_S2_ values in both species, and single-bends (i.e. stage 2 absent) were reported in three cases in sicklefin lemon sharks. This plasticity in stage 2 might confer an energetic advantage to the prey when responding to a predatory threat under different contexts. For example, when the danger is not imminent, a low-energy response may be sufficient to avoid predation.

Work on teleosts indicates that the use of a two-gear, fast or slow, response system may be another way of energy saving depending on the strength of the perceived threat (Domenici and Hale, 2019). Slow or fast escape responses depend on the stage 1 turning rates (ω_S1_ or ω_Mean_), which are driven by different muscle contraction speeds and/or neural pathways (Domenici and Batty, 1994; Nissanov et al., 1990). Domenici et al. (2004) found that *S. suckleyi* displayed both slow and fast escape responses, based on bimodal distributions of ω_S1_ and on ω_Mean_ and significantly different slopes found for the correlation between θ_S1_ and *T*_S1_ for fast and slow responses. This indicates that sharks can also take advantage of such a fast-or-slow system. In our study, ω_S1_ did not show a clear bimodal distribution. The kernel density smooth applied to the distribution of ω_S1_ did not show signs of multimodality, not even when single-bends were included in the data for sicklefin lemon sharks (Fig. S4). The correlation between θ_S1_ and *T*_S1_ showed a single slope with no distinct groups deviating from the rest of the data. Experimentally, the occurrence of fast or slow responses may be determined by the distance or speed of the mechanical stimuli (Bhattacharyya et al., 2017; Domenici and Batty, 1997). These two factors were maintained within a small range in our study (see Materials and Methods). Therefore, our results suggests that tropical neonate sharks did not respond with a clear fast-or-slow system, at least under the experimental conditions we used. Notably, because the faster turns observed were approximately twice as fast as the slowest turns, as found in the fast versus slow escapes of *S. suckleyi* (Domenici et al., 2004), and because of the low number of single-bends, we cannot discard the possibility of a fast-or-slow system in tropical neonate sharks. Slow responses would be advantageous when responding to low-risk threats in order to minimize the energetic costs associated with escape responses.

The level of threat is likely very high in early stages of life but may vary among shark species. Although both species studied here showed no differences in escape latency (and were very low), the lower responsiveness in sicklefin lemon sharks (64.6%), compared with blacktip reef sharks (100%), suggests that the level of perceived threat varies between them. In fact, behavioral trials showed that neonate sicklefin lemon sharks and grey reef sharks (*Carcharhinus amblyrinchos*) dominated blacktip reef sharks (O. C. Weideli, personal communication), indicating that the level of threat is much higher in blacktip reef sharks than in sicklefin lemon sharks. Furthermore, there are reports of giant trevally (*Caranx ignobilis*; ∼120 cm *L*_T_) ramming and mortally injuring adult blacktip reef sharks (∼200 cm *L*_T_; McPherson et al., 2012). Thus, especially during early life stages, blacktip reef sharks may experience a higher frequency of predator attacks and therefore may be more prone to engage in a high-energy response than the more dominant sicklefin lemon sharks. It is possible that, at this stage of life, blacktip reef sharks relay in an all-or-nothing strategy, which would explain why we did not observe a clear fast-or-slow system as in adult *S. suckleyi*.

Short-latency responses may signify a competitive advantage to small neonate sharks that likely rely on rapidly moving away from an approaching, larger predator. Neonate sharks were fast to react to the stimulus; their escape latency was shorter than previous observations on adult sharks (67 ms; Schakmann et al., 2021) and similar to those of many teleosts (Domenici and Hale, 2019). Many possible reasons could explain such short latencies when compared with previous work on elasmobranchs, and similar to latencies found in teleosts with M-cells (Domenici and Hale, 2019). One reason may be the relatively high temperature, as it is known that temperature affects latencies (Domenici et al., 2019), but other reasons may include species-specific differences and methods. Our methods, however, were similar to those of many previous studies that have tested for escape latency in teleosts. Given the importance of M-cells in minimizing escape latency in teleosts (Hecker et al., 2020), we cannot dismiss that neonate individuals of these tropical shark species may also possess M-cells. In fact, early ontogenetic stages of *S. acanthias* (embryos) and *Dalatias licha* show a transient Mauthner apparatus (Bone, 1977). Future anatomical studies would be necessary to test this hypothesis. An alternative explanation is that sharks use a different neural pathway to generate short-latency responses. Short-latency responses in teleosts can also be activated without M-cells (i.e. by other startle neurons, namely MiD2cm and MiD3cm; Liu and Fetcho, 1999). Regardless of whether M-cells are involved, a system that facilitates short latencies is fundamental to allow a small prey to rapidly change its direction, moving perpendicular to an approaching predator (Abrahams, 2006). Therefore, future studies that couple behavioural evidence with neural control in neonate sharks would be useful in order to understand the functional link between neural commands and escape performance in sharks.

This study improves our knowledge on escape behaviours by reporting, for the first time, detailed escape kinematics for neonate and tropical sharks. Laboratory studies impose logistical limitations on studying large taxa. Here, we successfully applied a common methodology to two shark species to understand behaviours that are important for fitness. We highlighted the main components of the escape response, such as high turning agility and fast reaction times, and their potential contribution toward survival. It is important to emphasize that future studies on the neuromuscular control of escape responses in sharks will be of particular value. Understanding how neonate sharks exploit coastal habitats where predation pressure is high, and how future environmental change will affect escape ability in sharks, is important. For instance, rising sea surface temperatures owing to anthropogenic climate change (Bindoff et al., 2019) are likely to alter predator–prey dynamics (Allan et al., 2015; Allan et al., 2017; Domenici et al., 2019). Hence, understanding escape behaviour and its physiological and morphological constraints in neonate sharks is essential for assessing how global change will shape future fish communities (Wheeler et al., 2020).

## Supplementary Material

10.1242/jexbio.243973_sup1 Supplementary information
