# Supplementary material for: Escape response kinematics in two species of tropical shark: short escape latencies and high turning performance
Source: J Exp Biol. 2022 Nov 18;225(22):jeb243973. doi: 10.1242/jeb.243973 (PMC9845744; doi:10.1242/jeb.243973)
Supplement: Supplementary information [file jexbio-225-243973-s1.pdf]

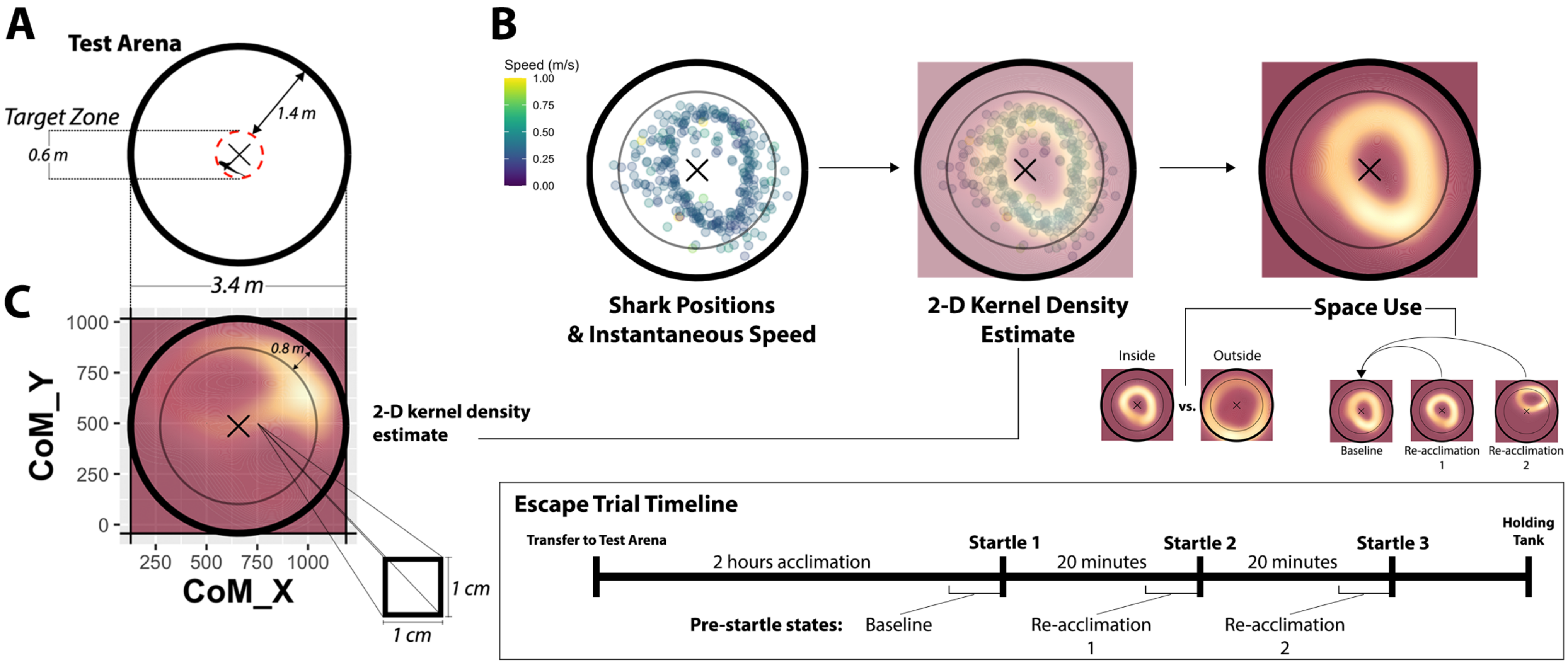

Fig. S1. Acclimation to test arena. We assessed the pre-startle state of each individual after 2 hours of un-disturbed swimming in the novel environment (the test arena, A, Baseline), and after 20 minutes of re-acclimation before the 2nd and 3rd startles (Re-acclimation 1 and 2, respectively). See escape trial timeline insert. Sharks were recorded for 5-minutes at 120 fps and manually tracked at 1 fps. First, we calculated the swimming speed (B). Low (≤ 1 m s^{-1}) and constant (no bursts) swimming speed characterized acclimated (or re- acclimated) individuals. Second, we calculated space use with a two-dimensional kernel density analysis of the tracking points (B, C). For this, the test arena was divided into two areas: a 1.85 m-diameter inner circle centred at the middle of the test arena (open space), and the remaining 0.8-m wide outer band ranging from the perimeter of the inner circle to the wall of the test arena (C). We used the function *kde2d* from the R package *MASS* (R Core Team, 2019; Venables and Ripley, 2002) on the tracking points to apply a two-dimensional kernel density estimation with an axis-aligned bivariate normal kernel, evaluated on a square grid with limits at the wall of the pool. Grid size was 1 cm^2, and bandwidth was chosen via the normal reference distribution (Venables and Ripley, 2002). The swimming pattern was analysed by classifying the resulting density points as inside (open space) or outside of the inner circle based on their *x* and *y* coordinates (Space Use in B). For simplicity, we report the percentage time spent in the open portion of the pool (inside). Baseline space use (before the 1st startle) was contrasted with the space use of Re-acclimation 1 and Re-acclimation 2 of each individual. Re-acclimated individuals resumed similar space use to their baseline.

Journal of Experimental Biology • Supplementary information

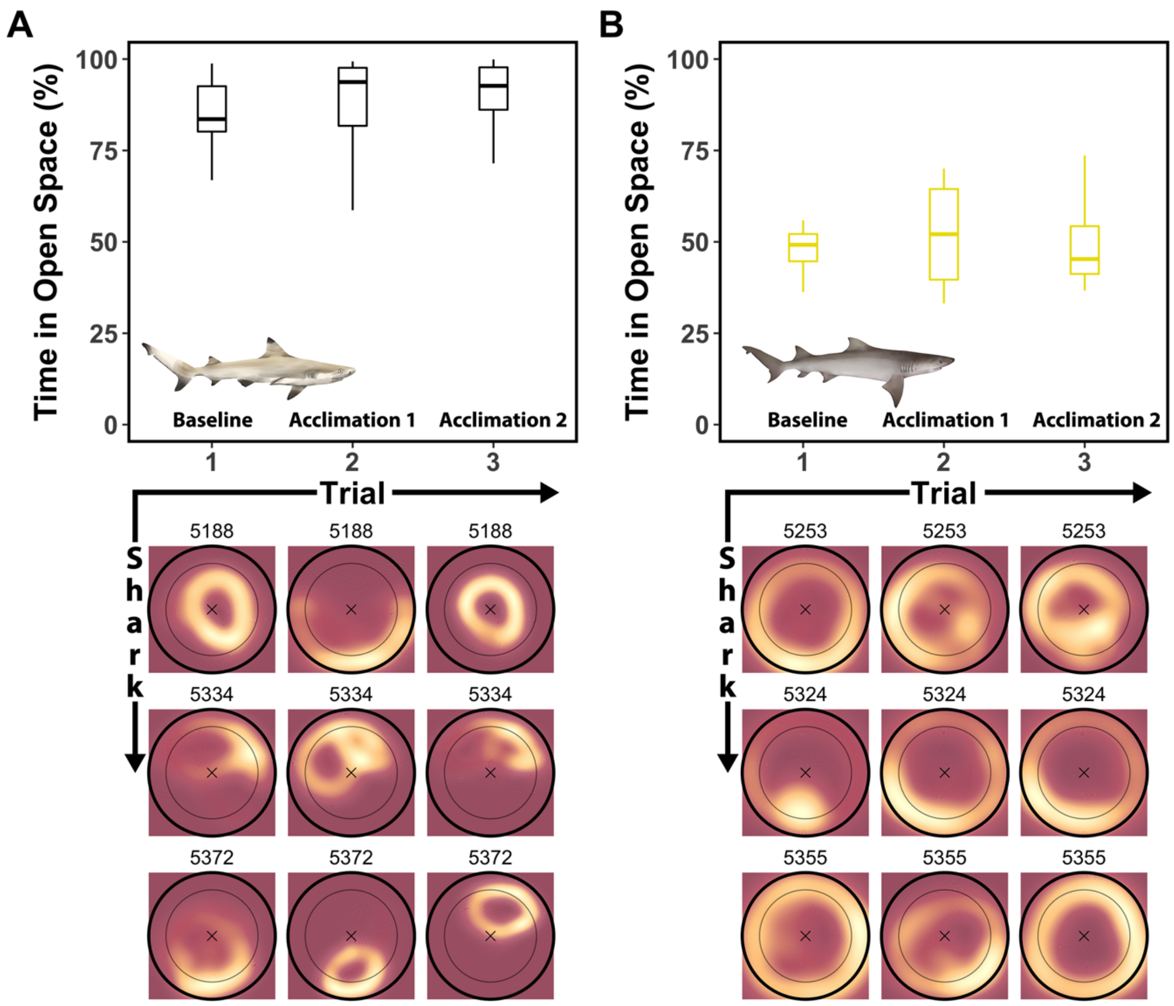

Fig. S2. Space use between pre-startle states. Percentage time in open space for (A) *Carcharhinus melanopterus* and (B) *Negaprion acutidens*. Boxplots showing median (solid black bar), first and third quartiles (left and right hinges, respectively), lowest and maximum values (left and right whiskers, respectively). Pre-startle state had no effect on space use in *C. melanopterus* (one-way ANOVA: $F(1, 29) = 1.17$, $p = 0.288$) or *N. acutidens* (one-way ANOVA: $F(1, 12) = 0.069$, $p = 0.798$). Below each boxplot are grid examples of three different sharks (vertical axis), for each species, for each pre-startle state/trial (horizontal axis). Grids are 2-D kernel density analysis of routine space use using the COM *x* and *y* coordinates over 5 minutes of tracking. Lighter colour indicates higher density. The inner circle (thin line) marks a 0.8-m space from the wall. Shark illustrations by Erin Walsh.

Journal of Experimental Biology • Supplementary information

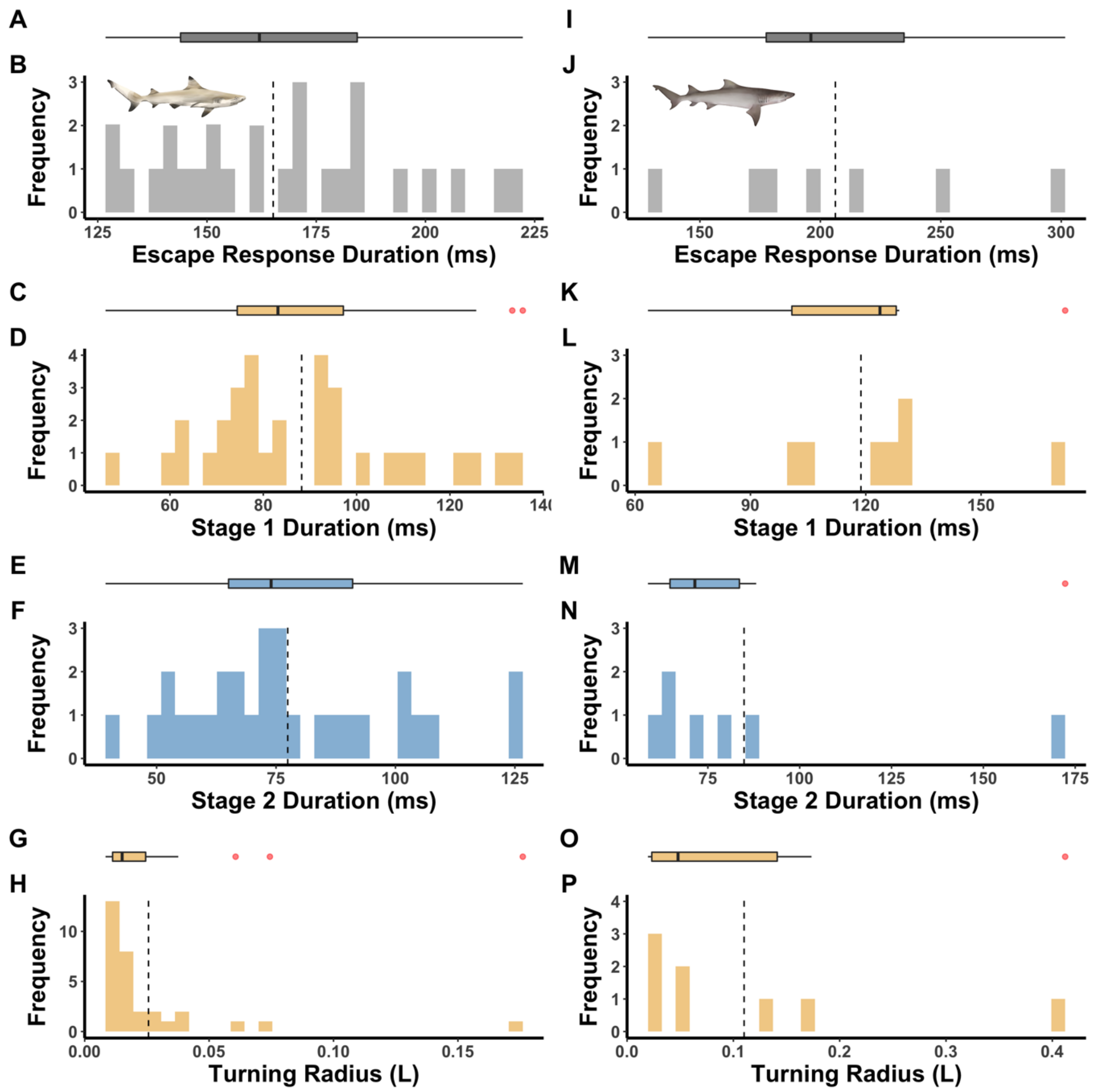

Fig. S3. Durations and turning radius for *Carcharhinus melanopterus* (left) and *Negaprion acutidens* (right) double-bends. Frequency distributions and associated boxplots for (A, B and I, J) escape response duration, (C, D and K, L) stage 1 duration, (E, F and M, N) stage 2 duration and (G, H and O, P) turning radius. Vertical black dashed lines are the means. Boxplot showing median (solid black bar), first and third quartiles (left and right hinges, respectively), lowest and maximum values (left and right whiskers, respectively), and extreme values (red dots). Durations are in milliseconds (ms) and turning radius in lengths (L). Grey: total escape response. Orange: stage 1. Blue: stage 2. Shark illustrations by Erin Walsh.

Journal of Experimental Biology • Supplementary information

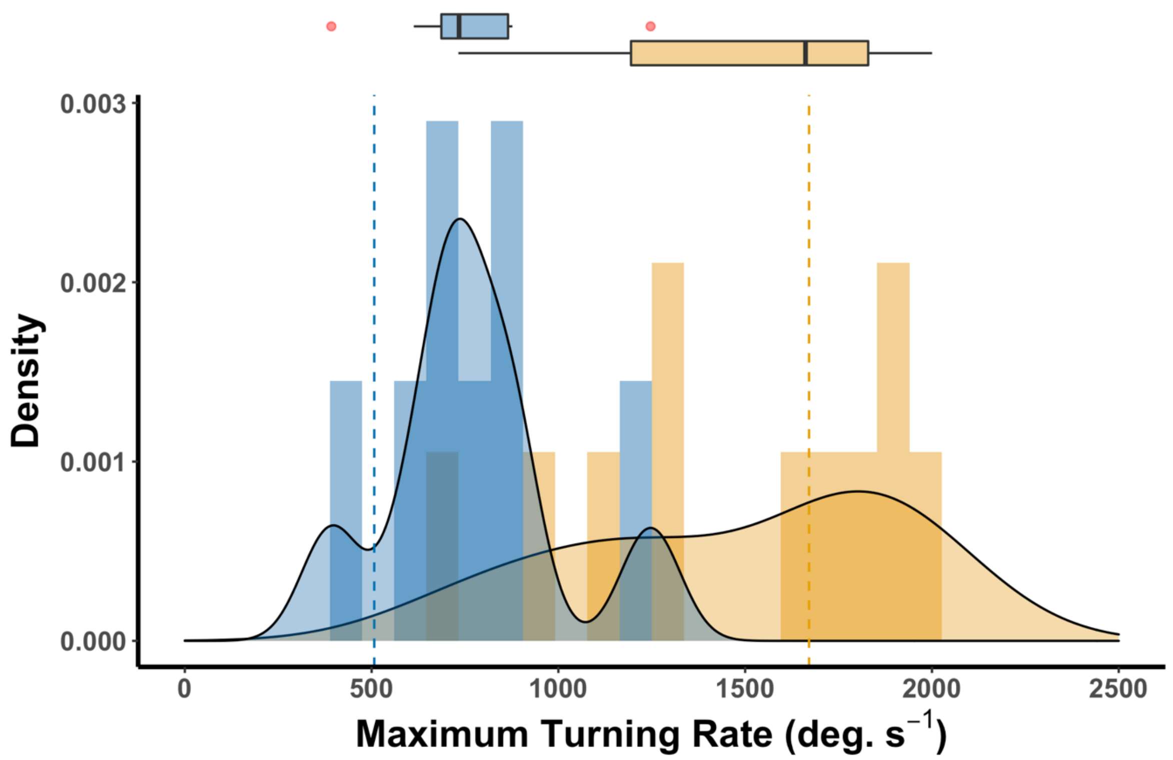

Fig. S4. Absolute maximum turning rate density distributions for double-bend and single-bend escape responses in *Negaprion acutidens*. Stage 1 is orange and stage 2 is blue. Smoothed distributions are kernel density estimates. Vertical dashed lines are means. Associated boxplots showing median (solid black bar), first and third quartiles (left and right hinges, respectively), and lowest and maximum values (left and right whiskers, respectively), and extreme values (red dots).

Journal of Experimental Biology • Supplementary information

Table S1. Locomotor and turning performance metrics of escape responses in sharks and similar-sized teleosts

Species	Life stage	Source	Experimental conditions			Response type	Motor pattern	*n*	Duration (ms)	Locomotor performance			Turning performance				Timing	
			Stimulus	L_T (cm)	Temperature (°C)					U_{max} (m s^{-1})	U_{max} (BL s^{-1})	α_{max} (m s^{-2})	θ_{S1} (deg)	ω_{Mean} (deg s^{-1})	ω_{S1} (deg s^{-1})	Turning radius (L)	Latency (ms)	Minimum latency (ms)
**Chondrichthyan escape responses**				Indicative ranges (Domenici and Hale, 2019):					30–200	1.5–2.8	3–30	20–150	0–180	500–8000		0.05–0.4	5–150	
Blacktip reef shark, *Carcharhinus melanopterus*	Neonate	Present study	Mechano-acoustic	57.3±0.53	29	Double-bend escape response	C-start	32 (12)	173.25±9.40 (*n*=30)	3.01±0.11	5.2	36.5±2.25	30.14–178.90	1148±32.55	1844±41.20	0.03±0.01 (*n*=31)	26.5±2.91 (*n*=30)	8.33
Sicklefin lemon shark, *Negaprion acutidens*			Mechano-acoustic	66.5±1.07	29	Double-bend escape response	C-start	8 (6)	206.23±19.25 (*n*=7)	2.70±0.14	4.1	34.3±5.80	33.83–138.28	972±99.07	1671±111	0.11±0.05	17.6±9.21 (*n*=9)	4.17
			Mechano-acoustic	65.3±2.42	29	Single-bend escape response	C-start	3 (3)	177.86±9.64	1.40±0.16	2.1	27.4±6.15	63.15–190.71	526±141.35	977±163	0.09±0.07		
Atlantic spiny dogfish, *Squalus suckleyi*	Adult	Domenici et al. (2004)	Manual thrust with a pole	58.6±4.6	12	Double-bend escape response (fast)	C-start	8 (5)	295.4±41.4	1.17±0.13	1.99	31.5±3.3	53.40–101.50[c] (72.2±7.8)	471±12	889±38	0.060±0.006	–	–
			Manual thrust with a pole	58.6±4.6	12	Double-bend escape response (slow)	C-start	7 (5)	281.5±34.9	0.80±0.07	1.36	19.4±1.5	26.30–74.22[c] (53.1±5.4)	268±14	499±20	0.074±0.007	–	–
		Schakmann et al. (2021)	Mechano-acoustic	76.3±6.1[a]	13.3	Not defined	C-start	11	–	–	–	–	–	–	–	–	97.8±18.2	66.7
**Teleost escape responses**																		
Pike, *Esox lucius*	Adult	Harper and Blake (1990)	Manual thrust with a pole	37.8±1.9	15–20	Potentially single- and double-bend responses combined (as Types I, II and III)	S-start followed by C-start	25 (4)	108±10.00	3.97±0.36	10.5	120.2	–	–	–	–	–	–
		Frith and Blake (1991)		41	10–14	Double-bend escape response	C-start	3 (1)	129.3	3.5	8.7	151.5	–	–	–	–	–	–
		Frith and Blake (1995)		39.5	8–12	Escape response (likely double-bend)	C-start	1 (1)	–	–	–	–	–	–	–	0.09[d]	–	–
Rainbow trout, *Oncorhynchus mykiss*		Webb (1976a,b)	Direct current electric shock	38.7	15	Escape response	C-start[b]	? (5)	100	2.8	7.4	40.6	–	–	–	0.180		
				34.7	15		C-start[b]	? (7)	100	1.8	5.3	34.6	–	–	–	0.150		
		Harper and Blake (1990)	Manual thrust with a pole	31.6±2.0	15–20	Potentially single- and double-bend responses combined (as Types I and II)	S-start followed by C-start	30 (8)	125	2.77	8.7	59.7	–	–	–	–	–	–

Duration is equivalent to T_{Escape} for double-bend escape responses and equivalent to T_{S1} in single-bends. For non-escape responses, angular velocity is listed under stage 1 maximum turning rate (ω_{S1}) as it represents the rate of turn of a first body bend during a yaw turn. Speed (U_{max}) in body lengths per second (BL s^{-1}) was calculated by diving the average U_{max} in m s^{-1} by the average L_T in metres if not already reported by the authors. See Table 1 for variable definitions. Values are means±s.e.m. otherwise stated. *n* is the number of turns analysed and the number of different individuals is in parentheses. Teleost escape responses were obtained from Domenici and Blake (1997).

[a]Standard deviation rather than standard error. [b]Reported as L-starts in the original article. [c]Range calculated from fig. 3 in Domenici et al. (2004). [k]Calculated from fig. 1B-ii in Frith and Blake (1995).

Journal of Experimental Biology • Supplementary information

Table S2. Locomotor and turning per–formance metrics of non-escape responses in sharks (continuation of Table S1)

Species	Life stage	Source	Experimental conditions			Response type	Motor pattern	*n*	Duration (ms)	Locomotor performance			Turning performance				Timing	
			Stimulus	L_T (cm)	Temperature (°C)					U_{max} (m s^{-1})	U_{max} (BL s^{-1})	α_{max} (m s^{-2})	θ_{S1} (deg)	ω_{Mean} (deg s^{-1})	ω_{S1} (deg s^{-1})	Turning radius (L)	Latency (ms)	Minimum latency (ms)
**Chondrichthyan non-escape responses**																		
Scalloped hammerhead shark, *Sphyrna lewini*	Juvenile	Kajiura et al. (2003)	Dipole electric field (prey simulation)	75.1±18.0[c]	–	Sharp[e] feeding turns	–	45 (20)	–	–	–	–	>90	–	469.83±48.70[g]	0.183	–	–
Sandbar shark, *Carcharhinus plumbeus*			Dipole electric field (prey simulation)	69.2±5.16[c]	–	Sharp[e] feeding turns	–	45 (20)	–	–	–	–	>90	–	246.40±6.90[g]	0.193	–	–
Bonnethead shark, *Sphyrna tiburo*		Hoffmann and Porter (2019)	No stimulus[a]	77.1–83.5	–	Routine turns	–	9 (4)	–	0.51±0.02[f]	0.76±0.03[f]	–	–	–	150.4	0.374[i]	–	–
Pacific spiny dogfish, *Squalus suckleyi*	Adult	Hoffmann et al. (2019)	No stimulus[a]	51.2–56.3[d]	–	Routine turns	–	9 (3)	–	–	–	–	–	–	92.03±15.46[h]	–	–	–
Leopard shark, *Triakis semifasciata*	Juvenile	Porter et al. (2011)	No stimulus[b]	25.3–48.5	–	Sharp[e] routine turns	–	9 (9)	–	0.25±0.02	0.78±0.086	–	–	–	300.23±24.75[g]	0.006±0.0016[i]	–	–
Blacktip reef shark, *C. melanopterus*	Neonate	Present study	No stimulus	58.4±0.70	29	Routine turn	–	19 (5)	409±27.9	–	–	–	44.6±2.93	116±9.83	–	–	–	–
Sicklefin lemon shark, *N. acutidens*				66.5±1.55			–	10 (8)	559±41.1	–	–	–	42.7±3.00	78.4±6.12	–	–	–	–

For non-escape responses, angular velocity is listed under stage 1 maximum turning rate (ω_{S1}) as it represents the rate of turn of a first body bend during a yaw turn. See more details in Table S1.

[a]Path obstruction, avoiding a startle response. [b]Sharks turned when encountered a wall without provocation. [c]Standard deviation rather than standard error. [d]Fork length. [e]Sharp turn was defined as a change in trajectory of >90 deg by Kajiura et al. (2003). [f]Measured at maximum total rotation. [g]Reported as radians per second in the original article, converted multiplying by 180/π. [h]Calculated from data obtained from fig. 3C in Hoffmann et al. (2019). [i]Calculated as the minimum instantaneous turning radius during the turn.

Journal of Experimental Biology • Supplementary information

Journal of Experimental Biology • Supplementary information

Table S3. Fast-start kinematics for neonate blacktip reef (*Carcharhinus melanopterus*) and sicklefin lemon shark (*Negaprion acutidens*) escape responses.

Trial	Shark ID	Species	Response Type		Sex	L_T (cm)	Mass (kg)	θ_{S1} (deg.)	θ_{S2} (deg.)	T_{S1} (ms)	T_{S2} (ms)	T_{Escape} (ms)	v (m s^{-1})	U_{Averg} (m s^{-1})	U_{MAX} (m s^{-1})	a_{MAX} (m s^{-2})	ω_{S1} (deg. s^{-1})	ω_{S2} (deg. s^{-1})	ω_{Mean} (deg. s^{-1})	Turning Radius (L)
1	4954	Carcharhinus melanopterus	C-START	DOUBLE	FEMALE	61.2	1.24	57.53	-43.22	75.52	124.16	199.68	2.51	1.89	2.90	30.40	1439.14	-1123.86	820.79	0.12
2	4954	Carcharhinus melanopterus	C-START	DOUBLE				178.93	-14.31	133.93	51.39	185.31	2.05	1.65	3.11	22.25	1973.79	-605.34	1344.15	0.05
1	5027	Carcharhinus melanopterus	C-START	DOUBLE	FEMALE	55.4	0.9	97.73	-26.56	84.31	59.69	144.00	3.68	2.11	3.22	44.26	2116.76	-1128.30	1193.02	0.19
2	5027	Carcharhinus melanopterus	C-START	DOUBLE				110.33	-14.27	92.00	39.76	131.77	2.61	1.84	3.14	32.65	2168.93	-546.75	1328.04	0.02
2	5170	Carcharhinus melanopterus	C-START	DOUBLE	FEMALE	59.6	0.98	73.31	-39.10	76.80	106.24	183.04	3.48	2.58	3.45	42.89	2140.61	-1228.13	1127.52	0.03
3	5170	Carcharhinus melanopterus	C-START	DOUBLE				59.76	-30.31	67.82	102.74	170.56	3.43	2.15	3.29	37.78	1608.93	-1193.09	1009.01	0.03
1	5186	Carcharhinus melanopterus	C-START	DOUBLE	MALE	59.4	1.04	155.12	-27.18	121.60	72.53	194.14	2.32	1.73	2.99	21.74	1901.22	-858.07	1504.82	0.04
2	5186	Carcharhinus melanopterus	C-START	DOUBLE				59.10	-41.67	74.56	86.87	161.43	3.07	2.11	2.73	55.27	1704.31	-1514.50	1037.04	0.04
3	5186	Carcharhinus melanopterus	C-START	DOUBLE				65.48	-34.81	78.56	75.36	153.92	3.78	2.07	2.80	58.19	1706.96	-1125.49	1064.23	0.04
1	5188	Carcharhinus melanopterus	C-START	DOUBLE	MALE	59.6	0.99	30.14	-37.21	47.49	79.93	127.43	3.44	1.87	2.92	39.02	1208.17	-1439.11	697.68	0.02
2	5188	Carcharhinus melanopterus	C-START	DOUBLE				77.19	-33.27	78.51	71.49	150.00	3.25	2.08	3.24	35.00	1796.22	-1251.22	1141.29	0.04
3	5188	Carcharhinus melanopterus	C-START	DOUBLE				44.87	-49.16	59.11	109.16	168.27	3.02	2.08	3.08	36.50	1569.17	-1506.13	967.53	0.04
2	5196	Carcharhinus melanopterus	C-START	DOUBLE	MALE	59.6	1.08	58.17	-40.98	71.42	89.77	161.19	3.45	2.18	3.03	34.64	1481.68	-1267.04	901.51	0.02
3	5196	Carcharhinus melanopterus	C-START	DOUBLE				79.83	-30.37	95.43	124.20	219.63	2.76	2.25	5.69	32.69	1713.62	-1214.29	1118.34	0.04
1	5282	Carcharhinus melanopterus	C-START	DOUBLE	MALE	54.4	0.81	56.24	-31.81	62.63	64.92	127.55	3.70	1.96	3.07	36.22	1565.66	-1272.00	905.98	0.03
2	5282	Carcharhinus melanopterus	C-START	DOUBLE				65.06	-27.46	62.62	65.86	128.48	3.14	1.91	2.66	41.79	2079.96	-1148.77	1161.32	0.03
3	5282	Carcharhinus melanopterus	C-START	DOUBLE				74.60	-34.67	80.53	100.84	181.37	3.21	NA	NA	38.03	1812.44	-1059.26	976.35	0.14
1	5327	Carcharhinus melanopterus	C-START	DOUBLE	MALE	54.2	1.05	75.27	NA	100.22	50.01	150.23	NA	1.69	2.53	29.22	1880.89	NA	1236.42	0.02
2	5327	Carcharhinus melanopterus	C-START	DOUBLE				63.53	-32.28	71.35	68.83	140.18	3.52	1.95	3.34	34.46	1828.36	-1097.43	1137.55	0.04
3	5327	Carcharhinus melanopterus	C-START	DOUBLE				77.02	-35.00	77.19	73.27	150.46	3.60	2.04	3.18	35.76	1994.65	-1326.78	1200.67	NA
1	5334	Carcharhinus melanopterus	C-START	DOUBLE	FEMALE	60.8	1.14	95.24	-6.93	91.52	NA	NA	NA	1.90	2.94	45.37	1985.78	NA	1215.71	0.08
2	5334	Carcharhinus melanopterus	C-START	DOUBLE				152.16	-13.13	131.75	75.65	207.40	1.68	2.04	2.51	77.24	1898.64	-246.30	1155.68	0.09
3	5334	Carcharhinus melanopterus	C-START	DOUBLE				88.47	-1.48	95.60	NA	NA	NA	1.78	2.44	15.85	1743.39	NA	1040.97	0.04
1	5339	Carcharhinus melanopterus	C-START	DOUBLE	MALE	51.6	0.74	78.76	-28.75	82.10	67.66	149.76	3.23	2.09	3.06	37.27	1898.23	-1214.58	1052.09	0.03
2	5339	Carcharhinus melanopterus	C-START	DOUBLE				94.75	-9.18	92.62	NA	NA	NA	1.17	1.82	29.31	1911.71	NA	1294.42	0.03
3	5339	Carcharhinus melanopterus	C-START	DOUBLE				141.98	-23.36	111.52	74.31	185.83	2.69	1.83	3.08	26.57	2080.17	-936.44	1537.73	0.05
1	5372	Carcharhinus melanopterus	C-START	DOUBLE	MALE	56.8	0.88	125.39	-26.66	114.67	55.79	170.47	2.45	1.37	2.71	16.54	1926.60	-1007.24	1306.05	0.03
2	5372	Carcharhinus melanopterus	C-START	DOUBLE				165.83	-39.26	124.28	92.93	217.21	2.15	2.10	3.00	29.73	2291.44	-1409.12	1376.67	0.07
3	5372	Carcharhinus melanopterus	C-START	DOUBLE				96.55	-22.60	95.57	83.87	179.44	2.84	1.92	2.59	32.84	1888.54	-776.75	1184.04	0.09
1	5373	Carcharhinus melanopterus	C-START	DOUBLE	MALE	57.4	0.97	111.63	-27.57	105.97	64.66	170.63	2.94	1.70	3.08	24.47	2043.00	-1072.83	1243.87	0.03
2	5373	Carcharhinus melanopterus	C-START	DOUBLE				76.22	-24.19	74.85	62.25	137.10	3.56	2.12	3.04	59.49	1845.79	-972.93	1144.42	0.03
3	5373	Carcharhinus melanopterus	C-START	DOUBLE				86.87	-13.86	91.01	51.89	142.90	3.04	1.66	2.57	28.21	1793.96	-751.58	1296.89	0.02
1	5174	Negaprion acutidens	C-START	DOUBLE	FEMALE	69.8	1.49	72.75	NA	100.34	NA	NA	1.21	1.69	2.63	37.48	1774.96	-1246.83	757.21	0.05
2	5245	Negaprion acutidens	C-START	DOUBLE	FEMALE	64	1.07	130.83	13.57	121.50	58.90	180.40	1.10	1.80	3.17	29.24	1661.62	-728.76	1229.10	0.03
1	5253	Negaprion acutidens	C-START	**SINGLE**	FEMALE	63.4	1.16	63.15	NA	195.77	NA	NA	NA	1.91	3.01	31.77	1117.26	-392.25	597.53	0.23
2	5253	Negaprion acutidens	C-START	DOUBLE				33.83	13.06	66.51	130.13	130.00	1.89	1.07	1.20	18.72	914.41	NA	557.95	0.02
2	5297	Negaprion acutidens	C-START	DOUBLE	MALE	61.8	1.03	124.84	27.68	128.23	215.59	216.00	0.46	1.75	3.11	27.68	2000.53	-861.37	1053.27	0.40
3	5297	Negaprion acutidens	C-START	**SINGLE**				94.12	NA	175.07	NA	NA	NA	1.41	1.70	24.18	1284.96	NA	752.77	0.04
1	5324	Negaprion acutidens	C-START	DOUBLE	FEMALE	70	1.52	64.51	17.33	103.50	174.72	175.00	1.35	1.65	2.82	27.93	1271.64	-612.86	698.38	0.05
3	5324	Negaprion acutidens	C-START	DOUBLE				43.10	NA	162.73	NA	250.00	0.36	1.67	2.33	30.65	1915.33	-877.00	1329.19	0.03
2	5324	Negaprion acutidens	C-START	**SINGLE**				190.71	13.12	171.44	249.97	NA	NA	1.22	1.27	39.28	732.25	NA	266.31	0.01
2	5355	Negaprion acutidens	C-START	DOUBLE	MALE	68.6	1.47	NA	NA	129.71	195.45	195.00	0.96	1.33	2.35	17.40	1883.84	-711.36	1248.15	0.17
3	5355	Negaprion acutidens	C-START	DOUBLE				138.28	18.13	128.70	297.37	297.00	0.66	1.85	2.15	72.31	1744.06	-739.48	863.20	0.13

Table S4. Linear mixed-effects model results. $\hat{d}^2$: residual variance, $\tau 00$: random intercept variance, $\hat{\alpha}$: intercept, $\hat{\beta}$: slope, DF: degrees of freedom. The number of stimulation (3 levels) was included as a random effect in all models.

Model	Random Effects	Fixed Effects	Observations/Groups	DF	Log-Likelihood	*p*-value
**Blacktip Reef Shark (*Carcharhinus melanopterus*)**						
ESCAPE<br>Stage 1 Angle ~ Stage 1 Duration	$\hat{d}^2 = 132.93$<br>$\tau_{00} = 0.05$	$\hat{\alpha} = 19.27$<br>$\hat{\beta} = 0.04$	32/3	28	-122.90	0.000
ROUTINE<br>Stage 1 Angle ~ Stage 1 Duration	$\hat{d}^2 = 74.60$<br>$\tau_{00} = 0.56$	$\hat{\alpha} = 21.10$<br>$\hat{\beta} = 0.06$	19/3	15	-70.66	0.004
ESCAPE<br>Velocity ~ Stage 1 Angle	$\hat{d}^2 = 0.02$<br>$\tau_{00} = 0.00$	$\hat{\alpha} = 2.19$<br>$\hat{\beta} = -0.01$	28/3	24	6.51	0.000
ESCAPE<br>Velocity ~ Stage 2 Angle	$\hat{d}^2 = 0.13$<br>$\tau_{00} = 0.00$	$\hat{\alpha} = 0.66$<br>$\hat{\beta} = 0.02$	28/3	24	-16.23	0.005
ESCAPE<br>Velocity ~ Maximum Stage 1 Turning Rate	$\hat{d}^2 = 0.13$<br>$\tau_{00} = 0.00$	$\hat{\alpha} = 2.85$<br>$\hat{\beta} = -0.001$	28/3	24	-19.60	0.006
ESCAPE<br>Velocity ~ Maximum Stage 2 Turning Rate	$\hat{d}^2 = 0.11$<br>$\tau_{00} = 0.00$	$\hat{\alpha} = 0.34$<br>$\hat{\beta} = 0.001$	28/3	24	-17.40	0.000
**Sicklefin Lemon Shark (*Negaprion acutidens*)**						
ESCAPE<br>Stage 1 angle ~ Stage 1 duration	$\hat{d}^2 = 135.27$<br>$\tau_{00} = 0.63$	$\hat{\alpha} = -100.62$<br>$\hat{\beta} = 1.71$	8/3	4	-30.11	0.001
ROUTINE<br>Stage 1 angle ~ Stage 1 duration	$\hat{d}^2 = 67.46$<br>$\tau_{00} = 0.01$	$\hat{\alpha} = 19.27$<br>$\hat{\beta} = 0.04$	10/3	6	-35.33	0.094
